# Neutralising antibodies block the function of Rh5/Ripr/CyRPA complex during invasion of *Plasmodium falciparum* into human erythrocytes

**DOI:** 10.1111/cmi.13030

**Published:** 2019-04-24

**Authors:** Julie Healer, Wilson Wong, Jennifer K. Thompson, Wengqiang He, Richard W. Birkinshaw, Kazutoyo Miura, Carol A. Long, Vladislav Soroka, Teit Max Moscote Søgaard, Thomas Jørgensen, Willem A. de Jongh, Christopher Weir, Ella Svahn, Peter E. Czabotar, Wai‐Hong Tham, Ivo Mueller, Paul N. Barlow, Alan F. Cowman

**Affiliations:** ^1^ Infection and Immunity Walter and Eliza Hall Institute of Medical Research Melbourne Victoria Australia; ^2^ Department of Medical Biology University of Melbourne Melbourne Victoria Australia; ^3^ Laboratory of Malaria and Vector Research National Institute of Allergy and Infectious Diseases, National Institutes of Health Bethesda Maryland USA; ^4^ ExpreS^2^ion Biotechnologies Horsholm Denmark; ^5^ Schools of Chemistry and Biological Sciences University of Edinburgh Edinburgh Scotland, UK

## Abstract

An effective vaccine is a priority for malaria control and elimination. The leading candidate in the *Plasmodium falciparum* blood stage is PfRh5. PfRh5 assembles into trimeric complex with PfRipr and PfCyRPA in the parasite, and this complex is essential for erythrocyte invasion. In this study, we show that antibodies specific for PfRh5 and PfCyRPA prevent trimeric complex formation. We identify the EGF‐7 domain on PfRipr as a neutralising epitope and demonstrate that antibodies against this region act downstream of complex formation to prevent merozoite invasion. Antibodies against the C‐terminal region of PfRipr were more inhibitory than those against either PfRh5 or PfCyRPA alone, and a combination of antibodies against PfCyRPA and PfRipr acted synergistically to reduce invasion. This study supports prioritisation of PfRipr for development as part of a next‐generation antimalarial vaccine.

## 1 INTRODUCTION

Over half the world's population remains at risk from malaria, with control efforts stalling and even being reversed in some of the highest burden countries (WHO, 2016). The development of an effective vaccine is a strategic priority in the global elimination of this disease (Moorthy, Newman, & Okwo‐Bele, 2013). *Plasmodium falciparum*, the causative agent of the most severe form of malaria, has a complex life cycle and is transmitted to humans by *Anopheles* mosquitoes. Sporozoites injected into the skin infect hepatocytes in the liver in a clinically silent pre‐erythrocytic phase of development. This phase is followed by a release of hepatic merozoites into the blood stream. These invade erythrocytes and perpetuate the pathogenic asexual blood stage causing malaria.

The complexity of its lifecycle together with high levels of sequence diversity in some of the early vaccine candidates (Conway, 2015) has challenged vaccine development. The only candidate to make it to Phase III clinical trials is the RTS,S/AS01 vaccine that targets the circumsporozoite antigen present on the pre‐erythrocytic stage of *P. falciparum* (Rts et al., 2012). This vaccine provides only moderately protective efficacy with a duration of 2 years or less in the target population of infants and children under 5 years of age (Greenwood & Doumbo, 2016). Strategies are being designed to develop a more effective vaccine. One approach is to include multiple antigens from different lifecycle stages, with a focus on those known to be essential for parasite growth and for which antibodies block development and onward transmission. A leading candidate antigen is *P*. *falciparum* reticulocyte binding protein homologue 5 (PfRh5; Payne et al., 2017).

PfRh5 is an essential protein for merozoite invasion that binds to basigin, its receptor on the surface of the erythrocyte membrane (Baum et al., 2009; Crosnier et al., 2011). The structure of PfRh5, both alone and in complex with basigin, has been determined by X‐ray crystallography (Chen et al., 2014; Wright et al., 2014). In the parasite, PfRh5 forms a trimeric complex with the cysteine‐rich protective antigen (CyRPA) and PfRh5‐interacting protein (PfRipr) during merozoite invasion (Chen et al., 2011; Reddy et al., 2015; Volz et al., 2016). The structure of CyRPA has also been solved both by itself and in complex with invasion inhibitory monoclonal antibodies (mAbs), revealing a six‐bladed β‐propeller fold (Chen et al., 2017; Favuzza et al., 2017). The crystal structure of PfRipr in isolation has not been solved; however, the structure of the PfRh5/CyRPA/PfRipr complex has recently been determined by cryo‐electron microscopy (Wong et al., 2019). The trimer forms an extended structure with CyRPA at its centre binding through opposing faces to PfRipr and PfRh5. Formation of this complex mediates its efficient binding via PfRh5 to basigin, and this initiates insertion of PfRh5 and PfRipr into the erythrocyte membrane, a pivotal step in the function of the PfRh5/CyRPA/PfRipr complex (Wong et al., 2019).

Both CyRPA and PfRipr are also essential for merozoite invasion, functioning at the same stage in this process as PfRh5 (Volz et al., 2016). Both proteins are housed within merozoites in micronemes (Chen et al., 2011; Volz et al., 2016), subcellular apically located organelles containing mutiple invasion‐related proteins. Upon erythrocyte contact and via a signalling mechanism that has yet to be fully understood, micronemes release their contents, allowing the PfRh5/CyRPA/PfRipr complex to form at the junction between the apical end of the invading merozoite and the erythrocyte membrane (Volz et al., 2016).

The rationale for developing an invasion‐blocking vaccine that targets members of this protein complex comes from studies of mouse, rabbit, and human and non‐human primate responses. Antibodies to recombinant PfRh5 and basigin, as well as soluble basigin itself, block the function of PfRh5 consequently inhibiting merozoite invasion (Bustamante et al., 2013; Crosnier et al., 2011; Douglas et al., 2011). Rabbit and mouse antibodies raised to recombinant CyRPA and PfRipr also inhibit the function of these proteins and block merozoite invasion (Chen et al., 2011, 2017; Douglas et al., 2015; Favuzza et al., 2017; Reddy et al., 2014). Furthermore, vaccination of *Aotus* monkeys with PfRh5 protected individuals against challenge with a heterologous strain of *P. falciparum* (Douglas et al., 2015). PfRh5 and PfRipr antibodies from malaria‐exposed individuals in various endemic regions are associated with naturally acquired immunity to malaria (Chiu et al., 2014; Richards et al., 2013; Tran et al., 2014) and inhibit parasite growth (Patel et al., 2013; Tran et al., 2014). Antibody responses to PfRh5, elicited in nonexposed volunteers in a Phase 1a human clinical trial, likewise block merozoite invasion in vitro (Payne et al., 2017). Monoclonal antibodies to CyRPA provide protection against *P. falciparum* when passively transferred into a NOD/SCID IL2Rγ(null) mouse model engrafted with human erythrocytes (Dreyer et al., 2012).

A PfRh5‐based vaccine is leading the clinical development pathway (Hjerrild et al., 2016; Jin et al., 2018; Payne et al., 2017), whereas CyRPA is also in development for testing in Phase 1 clinical trials. But the third member of this essential complex, PfRipr, has not received the same attention. This study was primarily designed to address whether PfRipr warrants further preclinical development as a potential blood‐stage malaria vaccine, either alone or in combination with PfRh5 and/or CyRPA. To understand the molecular basis for antibody‐mediated growth inhibition, mAbs against PfRipr, PfRh5, and CyRPA were tested for parasite growth inhibition and for their effect on the assembly of the PfRh5/CyRPA/PfRipr complex (Chen et al., 2017; Douglas et al., 2014). Furthermore, we examined naturally acquired immunity to the PfRh5/CyRPA/PfRipr complex and its components and show that individuals in the study population with higher levels of antibodies to Ripr and Rh5 but not CyRPA were protected from clinical malaria.

## 2 RESULTS

### 2.1 Recombinant PfRipr forms a tripartite complex with CyRPA and PfRh5

**Figure 1:**
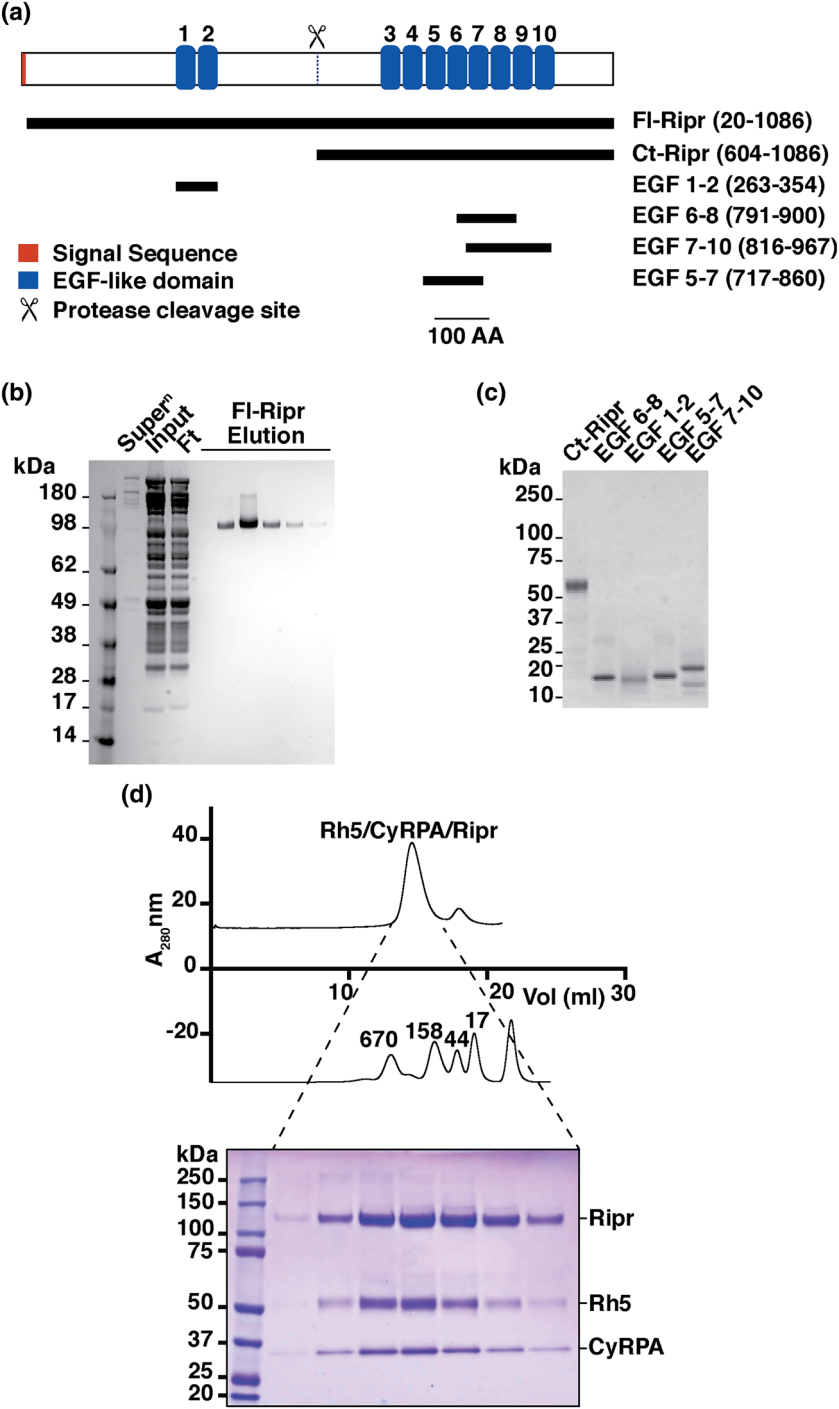
Domain structure of PfRipr and expression of recombinant proteins. (a) Domain structure of PfRipr showing the location of the EGF‐like domains (blue) and a protease processing site (scissors). Also shown are the boundaries of the recombinant proteins expressed to represent different domains of the PfRipr protein with residue range shown in brackets. (b) Expression and purification of Fl‐Ripr (amino acids 20–1086) from S2 Drosophila cells. Shown in the left panel is a SDS‐PAGE gel stained with Coomassie blue with molecular weight markers. Super^n^, culture supernatant; Input, concentrated supernatant loaded for affinity purification; Ft, flow through from affinity column; Elution, fractions eluted from affinity column. (c) Recombinant domains of PfRipr (as depicted in a) purified and stained with Coomassie blue. (d) SEC analysis of the PfRh5/CyRPA/PfRipr complex detected using Coomassie blue staining (lower panel)

PfRipr is a 120‐kDa protein with 87 cysteine residues. It consists of two regions that are separated by proteolytic cleavage both endogenously and in the recombinant expression system. The N‐terminal region contains two EGF‐like domains, whereas the C‐terminal region contains eight EGF‐like domains (Figure 1a; Chen et al., 2011). In order to analyse the function and immunogenicity of PfRipr, the full‐length mature protein (residues 20–1086 of the translated protein product) was expressed in Drosophila S2 cells (Fl‐Ripr) and purified from culture supernatant by affinity and size‐exclusion chromatography (SEC; Figure 1b). Additionally, specific domains of the PfRipr protein were expressed in either baculovirus‐infected Sf21 cells (Ct‐Ripr, residues 608–1086), *Escherichia coli* (EGF6‐8, residues 791–900), or *Pichia pastoris* (EGF7‐10, residues 816–961; EGF5‐7, 717–860; and EGF1‐2, 263–354; Figure 1a). Each of the purified proteins ran predominantly as a single band on SDS‐PAGE gels (Figure 1c).

To assess whether purified recombinant Fl‐Ripr was functional and capable of forming a complex with PfRh5 (Chen et al., 2011) and CyRPA (Chen et al., 2017), the proteins were incubated together and analysed by SEC (Figure 1d; Wong et al., 2019). This showed that the three proteins were present in approximately equimolar ratios in all fractions derived from the major peak, demonstrating the formation of a tripartite complex. This confirmed that the recombinant Fl‐Ripr was functional and conformationally intact by its ability to form a stable tripartite complex with PfRh5 and CyRPA (Wong et al., 2019).

### 2.2 Monoclonal antibodies that bind PfRipr inhibit P. falciparum growth

**Figure 2:**
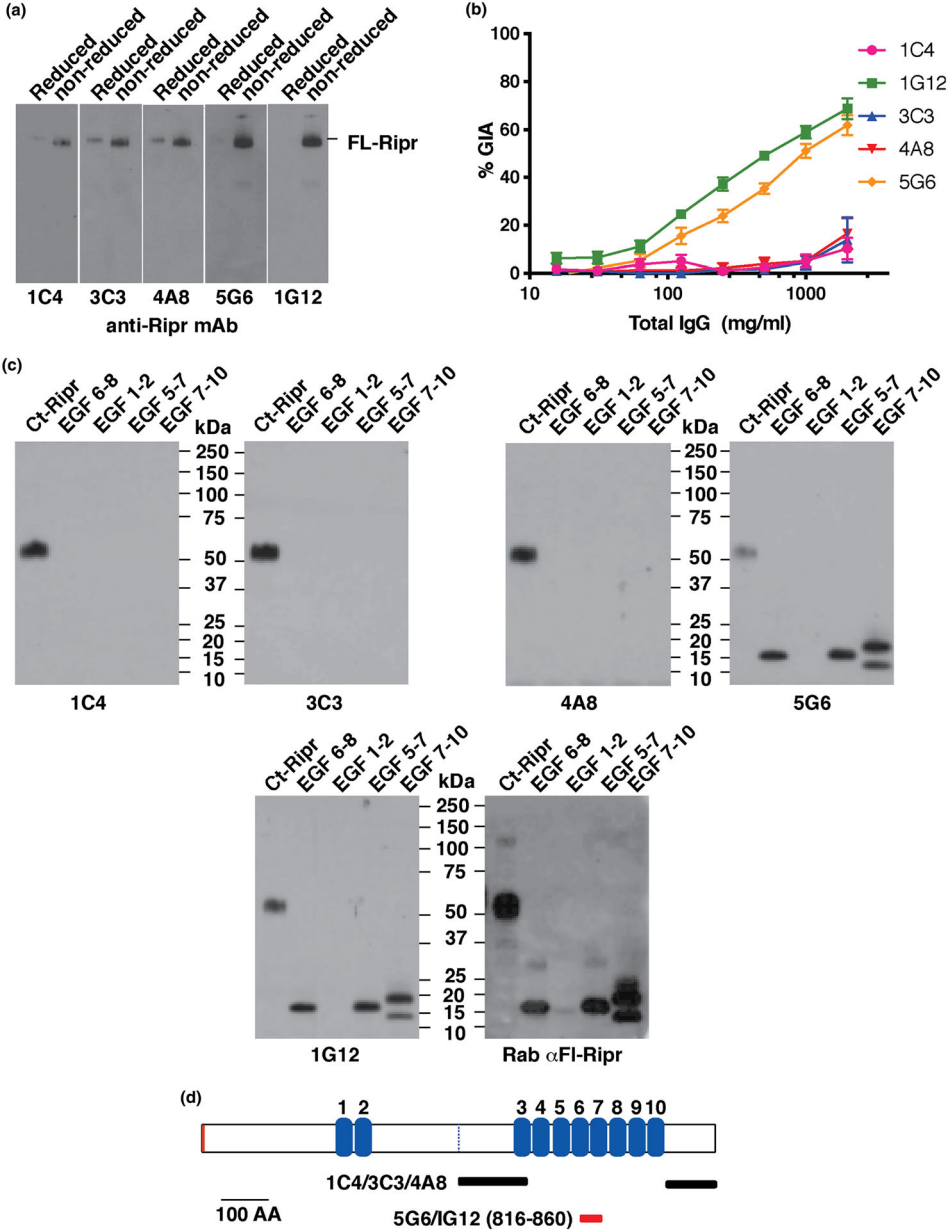
Identification of invasion neutralising monoclonal antibodies and mapping of epitopes on PfRipr. (a) Immunoblots of FL‐Ripr under reduced (R) and nonreduced (NR) conditions after SDS‐PAGE using monoclonal antibodies raised to CT‐Ripr. Monoclonal antibodies used were 1C4, 3C3, 4A8, 5G6, and 1G12. (b) Growth inhibition assays (GIA) of the anti‐Ripr monoclonal antibodies using 3D7 Plasmodium falciparum parasites. (c) Immunoblots of PfRipr recombinant protein domains to map the monoclonal antibody epitopes (1C4, 3C3, 4A8, 5G6, and 1G12). The final panel was screened using rabbit anti‐Fl‐Ripr polyclonal antibodies. (d) Domain structure of PfRipr and mapping of the spatial location of monoclonal antibody epitopes

Polyclonal antibodies to short regions of PfRipr were shown previously to inhibit *P. falciparum* growth (Chen et al., 2011). In order to understand the molecular mechanism involved, we derived mAbs to Ct‐Ripr (Figure 1a). Five mAbs (1C4, 3C3, 4A8, 5G6, and 1G12) were analysed by immunoblotting to determine their reactivity to Fl‐Ripr (Figure 2a). All mAbs bound to Fl‐Ripr under nonreducing conditions. Reactivity, however, was significantly decreased under reducing conditions and reduced Fl‐Ripr proved undetectable by 1G12 (Figure 2a).

We next used growth‐inhibition assays (GIA) to determine whether these mAbs had an effect on the ability of *P. falciparum* to invade and grow in human erythrocytes (Figure 2b). Although 1C4, 3C3, and 4A8 did not have any significant effect on parasite growth, 5G6 and 1G12 showed significant inhibition, with IC_50_ values of approximately 0.8 and 0.35 mg/ml, respectively. The level of inhibition with these mAbs is comparable to that reported in published studies with mAbs raised against Rh5 (Douglas et al., 2014).

To determine the location of the epitopes within PfRipr, the mAbs were tested by immunoblot against specific recombinant domains of the protein under nonreducing conditions (Figure 2c). The noninhibitory mAbs 1C4, 3C3, and 4A8 reacted with only Ct‐Ripr and did not bind to EGF 6‐8, EGF 1‐2, EGF 5‐7, or EGF 7‐10. Therefore, 1C4, 3C3, and 4A8 most likely bind to epitopes outside EGF 4‐10 but within the Ct‐Ripr domain (Figure 2d). The neutralising mAbs 5G6 and 1G12 reacted with nonreduced Fl‐Ripr, Ct‐Ripr, EGF 6‐8, EGF 7‐10, and EGF 5‐7 but not to EGF 1‐2. This finding was therefore consistent with the epitopes for both 5G6 and 1G12 being contained within domain EGF 7 of PfRipr (Figure 2d, red bar). The data were consistent with 1G12 and 5G6 binding to the same or overlapping epitopes. Similarly, 1C4, 3C3, and 4A8 may also bind to different but overlapping epitopes within the the C‐terminal region of PfRipr (Figure 2d, indicated as grey bars).

### 2.3 Kinetic properties of monoclonal antibodies that bind to PfRipr and CyRPA

In order to determine if the anti‐PfRipr monoclonal antibodies bound to overlapping epitopes, we used surface plasmon resonance (SPR) to measure their kinetic properties and competition studies to understand if they bound to spatially related areas of the Fl‐Ripr protein. Amine coupling mAb 1C4 to the chip did not provide a response in SPR experiments, likely due to the presence of a lysine in one of the complementary‐determining regions interfering with the SPR sensor surface blocking interactions with the antigen, and kinetic data were not obtained for this antibody. The equilibrium dissociation constants (*K*_*D*_) measured for 1G12, 5G6, 4A8, and 3C3 were between 4 × 10^−11^ and 2.4 × 10^−10^M showing these mAbs have a high affinity for their specific epitope (Figure S1A). However, the neutralising antibodies 1G12 and 5G6 have a threefold to sixfold tighter affinity than 4A8 and 3C3 (nonneutralising) driven by a tenfold to 20‐fold decrease in off rate. The comparable on and off rates for the kinetic dissociation (*k*_d_) and association (*k*_a_) constants for 4A8 and 3C3 are suggestive that they may bind a common epitope. The nonneutralising mAbs had a significantly faster off rate compared with the neutralising antibodies.

To determine which PfRipr mAbs bound to overlapping epitopes, competition assays were performed using SPR. MAbs 1G12 and 5G6 efficiently competed with each other and similarly 4A8, 3C3, and 1C4 also showed competitive binding to Fl‐Ripr (Figure S2). In contrast, 1G12 did not compete for binding to Fl‐Ripr with 4A8 or 3C3. Similar results were obtained for 5G6 (Figure S3). The competitive binding of 1G12 and 5G6 for binding to Fl‐Ripr is consistent with these mAbs binding to overlapping epitopes within the EGF 7 domain of Fl‐Ripr. Similarly, the properties of 4A3, 3C3, and 1C4 suggest that they bind to an overlapping epitope in a distinct region of the Ct‐Ripr domain (Figure 2d).

For comparison with the anti‐PfRipr antibodies, the kinetic parameters of mAbs raised previously to CyRPA, which we had shown to block growth of *P. falciparum* parasites (Chen et al., 2017), were measured (Figure S1B). The anti‐CyRPA mAbs 8A7, 5B12, and 3D1 inhibit *P. falciparum* growth as efficiently as the anti‐PfRipr monoclonal antibody 5G6 (Chen et al., 2017). The *K*_D_ values for the CyRPA mAbs ranged from 5.7 × 10^−10^ to 2.7 × 10^−9^M, which was weaker than those measured for the PfRipr neutralising antibodies. The *k*_*a*_ values for 8A7, 5B12, and 3D1 were similar, whereas the *k*_*d*_ values varied substantially, from 9.1 (±3.7) × 10^−5^ s^−1^ to 1.28 (±0.37) × 10^−4^ s^−1^.

We additionally used SPR to determine if the CyRPA mAbs bind overlapping epitopes. 3D1, 5B12, and 8A7 all showed competitive inhibition of binding, confirming that they bound to spatially similar epitopes on the CyRPA protein (Figures S1C and S4). As a control, we showed that the anti‐PfRipr antibody 3C3 shows no competitive inhibition against 8A7, 5B12, and 3D1 for binding to CyRPA. These competition SPR data are summarised in Table S1.

### 2.4 Neutralising antibodies can block formation of the PfRh5/CyRPA/PfRipr complex

**Figure 3:**
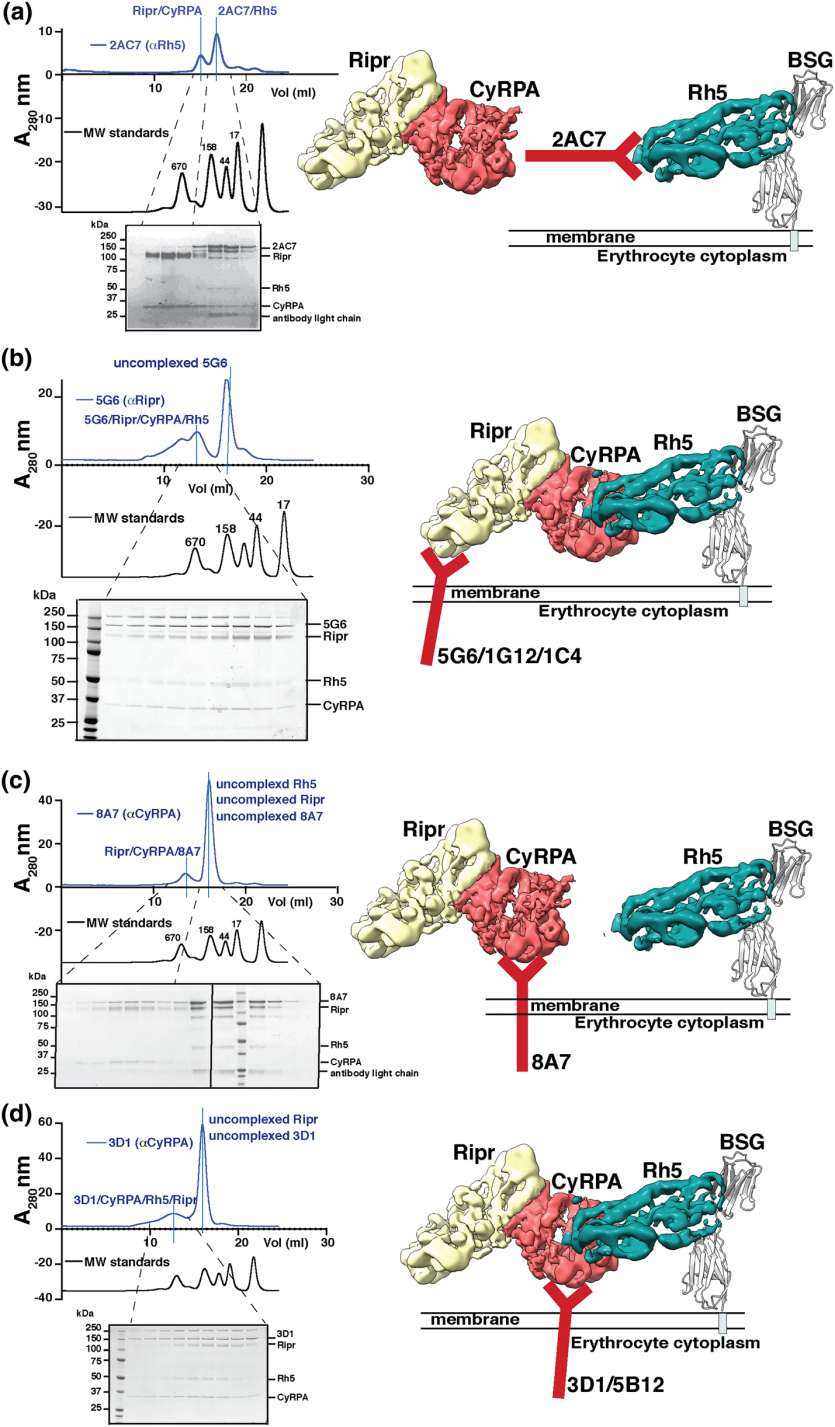
Neutralising monoclonal antibodies against PfRh5, PfRipr, and CyRPA and effect on PfRh5/CyRPA/PfRipr complex formation. (a) SEC analysis of inhibitory monoclonal antibody 2AC7 which binds to PfRh5 showing it blocks PfRh5/CyRPA/PfRipr complex formation. (b) SEC analysis of inhibitory monoclonal antibody 5G6 which binds to PfRipr but does not block PfRh5/CyRPA/PfRipr complex formation. Proteins are stained with Coomassie blue. SEC analysis for 1G12 and 1C4 are shown in Figure S5. (c) SEC analysis of inhibitory monoclonal antibody 8A7 which binds to CyRPA and blocksPfRh5/CyRPA/PfRipr complex formation. (d) SEC analysis of inhibitory monoclonal antibody 3D1 which binds to CyRPA but does not block PfRh5/CyRPA/PfRipr complex formation. Proteins in all SDS‐PAGE gels are stained with Coomassie blue. SEC analysis for 5B12 is shown in Figure S5. Traces shown above SEC gel profiles in all panels give elution volumes of molecular weight standards in kDa. Models of mAb binding to antigens and their effect on complex formation are shown underneath respective SEC panels

To understand the molecular mechanism of inhibition of merozoite invasion by the neutralising monoclonal antibodies to PfRipr, PfRh5, and CyRPA, we determined if they could block formation of the PfRh5/PfRipr/CyRPA tripartite complex (Figure 3). The proteins were pre‐incubated together in the presence of the different mAbs and the complexes analysed using SEC (Figure 3). MAb 2AC7 binds to PfRh5 and has previously been shown to inhibit parasite growth in vitro, and its epitope was located to a position on PfRh5 distinct from the basigin binding region (Douglas et al., 2014). In SEC, assays that detect complex formation 2AC7 block the ability of PfRh5 to bind directly to the CyRPA/PfRipr bipartite complex (Figure 3a). The bipartite CyRPA and PfRipr complex was able to form, and a PfRh5/2AC7 antibody complex was also observed. Therefore, the 2AC7 anti‐PfRh5 monoclonal inhibits merozoite invasion through interference with formation of the PfRh5/CyRPA/PfRipr complex whereas other anti‐PfRh5 antibodies can block ligand‐receptor binding.

As a control, we tested the ability of the noninhibitory anti‐Ripr monoclonal antibody 1C4 and found that it did not block formation of the ternary PfRh5/CyRPA/PfRipr complex but instead bound to PfRipr forming a quaternary complex (Figure S5). Similarly, the anti‐PfRipr neutralising antibodies 5G6 and 1G12 did not block formation of the PfRh5/CyRPA/PfRipr complex but formed quaternary complexes (Figures 3b and S5). Therefore, the neutralising antibodies 5G6 and 1G12 seem not to inhibit merozoite invasion through blocking formation of the PfRh5/CyRPA/PfRipr complex but likely interfere with the downstream function of this tripartite complex.

We had previously shown that mAb 8A7 binds CyRPA and blocks interaction with PfRh5 (Chen et al., 2017). This was confirmed here because when we tested the ability of this antibody to interfere with formation of the PfRh5/CyRPA/PfRipr complex using SEC (Figure 3c), both the ternary PfRipr/CyRPA/8A7 antibody complex and free PfRh5 were detected. In contrast, the growth‐inhibitory CyRPA mAbs 3D1 (Figure 3d) and 5B12 (Figure S5) formed quaternary PfRh5/CyRPA/PfRipr/3D1 and PfRh5/CyRPA/PfRipr/5B12 complexes. That only 8A7 (and not 3D1 or 5B12) blocked participation of CyRPA in the PfRh5/CyRPA/PfRipr complex must be reconciled with SPR results showing that 8A7, 3D1, and 5B12 compete for the same or similar epitopes on CyRPA. The slower disassociation rate of 8A7 (9.1 × 10^−5^ s^−1^) compared with 3D1 (1.28 × 10^−4^ s^−^1) and 5B12 (5.9 × 10^−4^ s^−^1) may be crucial for its ability to block PfRh5 binding. Taken together, these results show that neutralising antibodies block merozoite invasion through inhibition of formation of the PfRh5/CyRPA/PfRipr complex as well as through other mechanisms.

### 2.5 Antibodies to PfRh5, CyRPA, PfRipr, and the PfRh5/CyRPA/PfRipr complex inhibit P. falciparum growth

**Figure 4:**
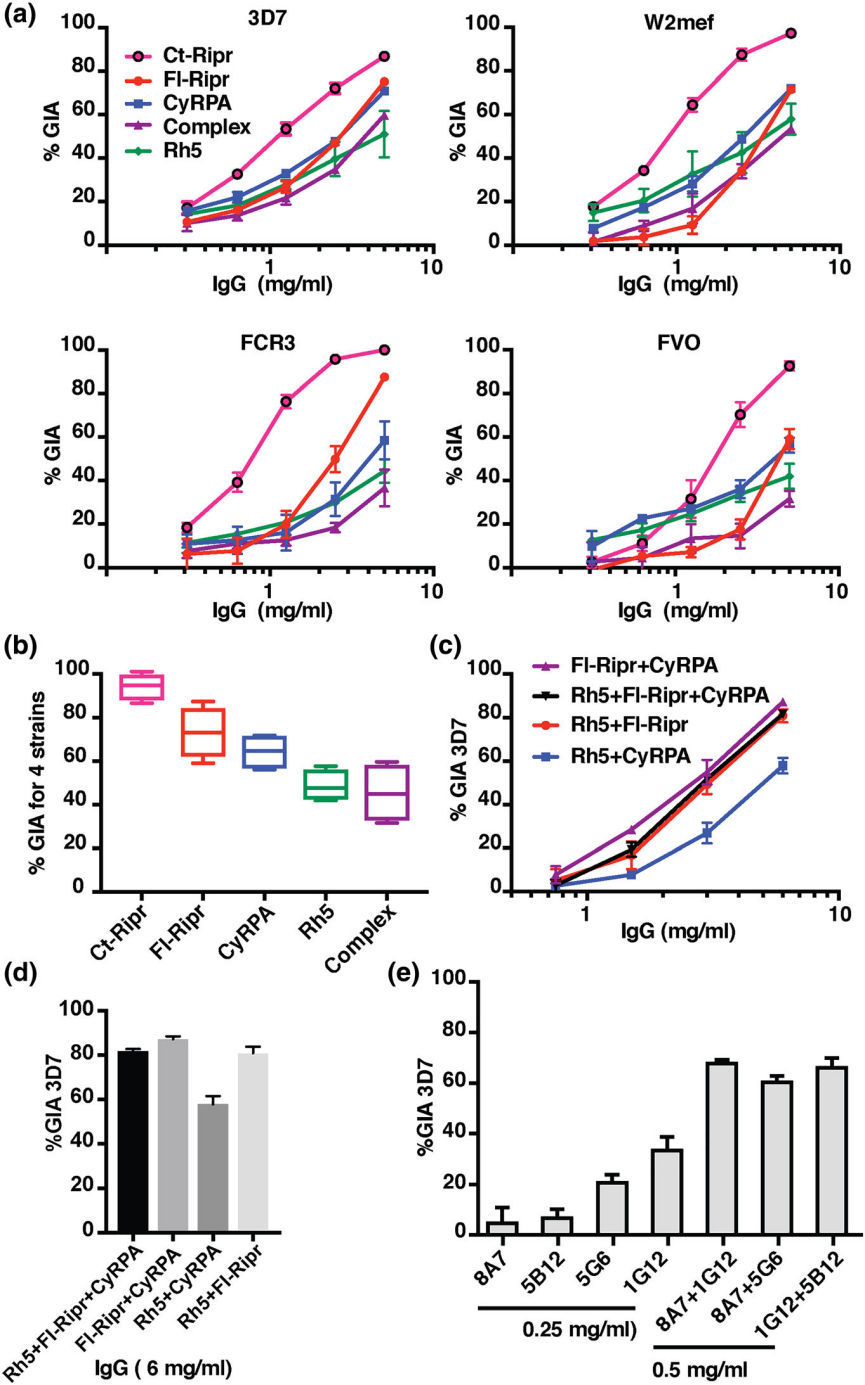
Antibodies against PfRh5, CyRPA, and PfRipr inhibit Plasmodium falciparum growth. (a) Growth inhibition assays using IgG rabbit antibodies raised to equal concentrations of Ct‐Ripr, Fl‐Ripr, CyRPA, PfRh5/CyRPA/PfRipr complex or PfRh5 with P. falciparum strains 3D7, W2mef, FCR3, and FVO. Plots are data from two biological replicates with mean and SEM shown. (b**)** Combined summary of GIA assays at 5 mg/ml IgG for 3D7, W2mef, FCR3, and FVO. (c) GIA assays using 3D7 parasites where anti‐Fl‐Ripr, CyRPA, and PfRh5 antibodies have been added in combinations. Total IgG concentration shown on x‐axis. (d) GIA assays using 3D7 parasites where polyclonal antibodies against Rh5, CyRPA and PfRipr have been combined to a total IgG concentration of 6 mg/ml. * Denotes significantly different activity p < .05 (t test). (e) Expected and actual level for inhibition of parasite growth when CyRPA and PfRipr monoclonal antibodies have been combined. Monoclonal antibodies against Ripr (1G12 and 5G6) were combined with those against CyRPA (5G6 and 5B12). Shown in black bars is the expected level of inhibition derived by adding the GIA activity of the individual mAbs each at a concentration of 0.25 mg/ml. In grey bars is the actual GIA activity of the combined mAbs at a total concentration of 0.5 mg/ml (0.25 mg/ml of each)

Polyclonal antibodies to the individual antigens PfRipr, PfRh5, and CyRPA were previously shown to inhibit merozoite invasion and consequently growth of *P. falciparum* in vitro (Baum et al., 2009; Patel et al., 2013; Reddy et al., 2014; Reddy et al., 2015; Weiss et al., 2015; Williams et al., 2012). However, it is unknown if antibodies raised to the PfRh5/CyRPA/PfRipr complex are more efficient than Abs raised against the components at inhibiting *P. falciparum* growth. To test this, in vitro growth‐inhibition assays were performed with the *P. falciparum* strains 3D7, W2mef, FCR3, and FVO (Figure 4a,b). Rabbits were immunised with the individual antigen components or equivalent amount of the assembled complex, where the components were in a 1:1:1 molar ratio. Antibodies to Ct‐Ripr resulted in significantly higher levels of growth inhibition for all *P. falciparum* strains tested with approximately 94.3% and antibodies against Fl‐Ripr resulted in 73.3% inhibition at 5 mg/ml (Figure 4a,b). In comparison, antibodies to CyRPA and PfRh5 were less inhibitory when compared across all four strains, at 64.4% and 46.8%, respectively. Consistent with the absence of significant polymorphism in these antigens in the *P. falciparum* population, antibodies to each of the individual proteins and the complex showed no significant difference in GIA against 3D7, W2mef, FCR3, or FVO. Antibodies raised to the PfRipr/CyRPA/PfRipr complex showed the lowest level of inhibition (Figure 4a), comparable with PfRh5 antibodies when compared across the four strains (Figure 4b).

This result suggested that the epitopes of some neutralising antibodies were buried in the PfRipr/CyRPA/PfRipr ternary complex and that immunizing with individual proteins may be a preferable option. To explore this possibility further, we tested the ability of combinations of antibodies raised to individual proteins to inhibit *P. falciparum* growth (Figure 4c,d). A combination of antibodies to Fl‐Ripr/CyRPA, PfRh5/Fl‐Ripr, and PfRh5/FL‐Ripr/CyRPA was all able to inhibit *P. falciparum* growth to approximately 70% at 6 mg/ml. In contrast, the combination of PfRh5/CyRPA antibodies was significantly less efficient in their ability to block parasite growth (*p* = .01). Taken together, these results show that antibodies raised to Fl‐Ripr inhibited *P. falciparum* growth more effectively than those raised against PfRh5 and CyRPA.

Comparative ELISA was performed to examine whether serum titres of antibodies correlated with GIA activity. In these assays, sera raised against the antigens in trimeric complex was tested alongside sera raised against the individual antigens with the individual antigen on the plate (Figure S6). This demonstrated that even when rabbits were immunised with excess antigen (200 μg per dose), the resulting antibody titres were always lower in the complex sera than when a single immunising antigen was used. This was most striking in the case of sera against CyRPA, where approximately 30‐fold lower titres were seen for CyRPA in complex with Ripr and Rh5 compared with CyRPA as a single immunogen. Titres against Rh5 were around tenfold lower in the sera raised against the Rh5/CyRPA/Ripr complex whereas titres against Ripr_FL in the complex were around threefold lower.

In further experiments, a combination of the neutralising monoclonal antibodies against PfRipr and CyRPA was tested for their ability to inhibit *P. falciparum* growth (Figure 4e). The mAbs 8A7 (anti‐CyRPA), 1G12 (anti‐PfRipr), and 5G6 (anti‐PfRipr) and their combinations 8A7/1G12, 8A7/5G6, and 1G12/5B12 were tested in GIA. Combinations of mAbs (0.5 mg/ml total, or 0.25 mg/ml each) were assessed in GIA. The percentage growth inhibition values were plotted alongside an “expected” level of inhibition obtained by summing growth‐inhibition percentages for the individual antibodies at 0.25 mg/ml each (Figure 4e). In all cases, the combinations brought about much greater levels of inhibition than the sum of the individual components, suggesting a synergistic effect. Overall, these results suggest that antibodies to PfRipr are more efficient at blocking *P. falciparum* growth than antibodies to PfRh5 or CyRPA, but combinations of antibodies to multiple components of the protein complex resulted in the highest inhibition values.

### 2.6 Associations between antibody responses to PfRh5, CyRPA, PfRipr, and the PfRh5/CyRPA/PfRipr complex and protection from clinical malaria

**Figure 5:**
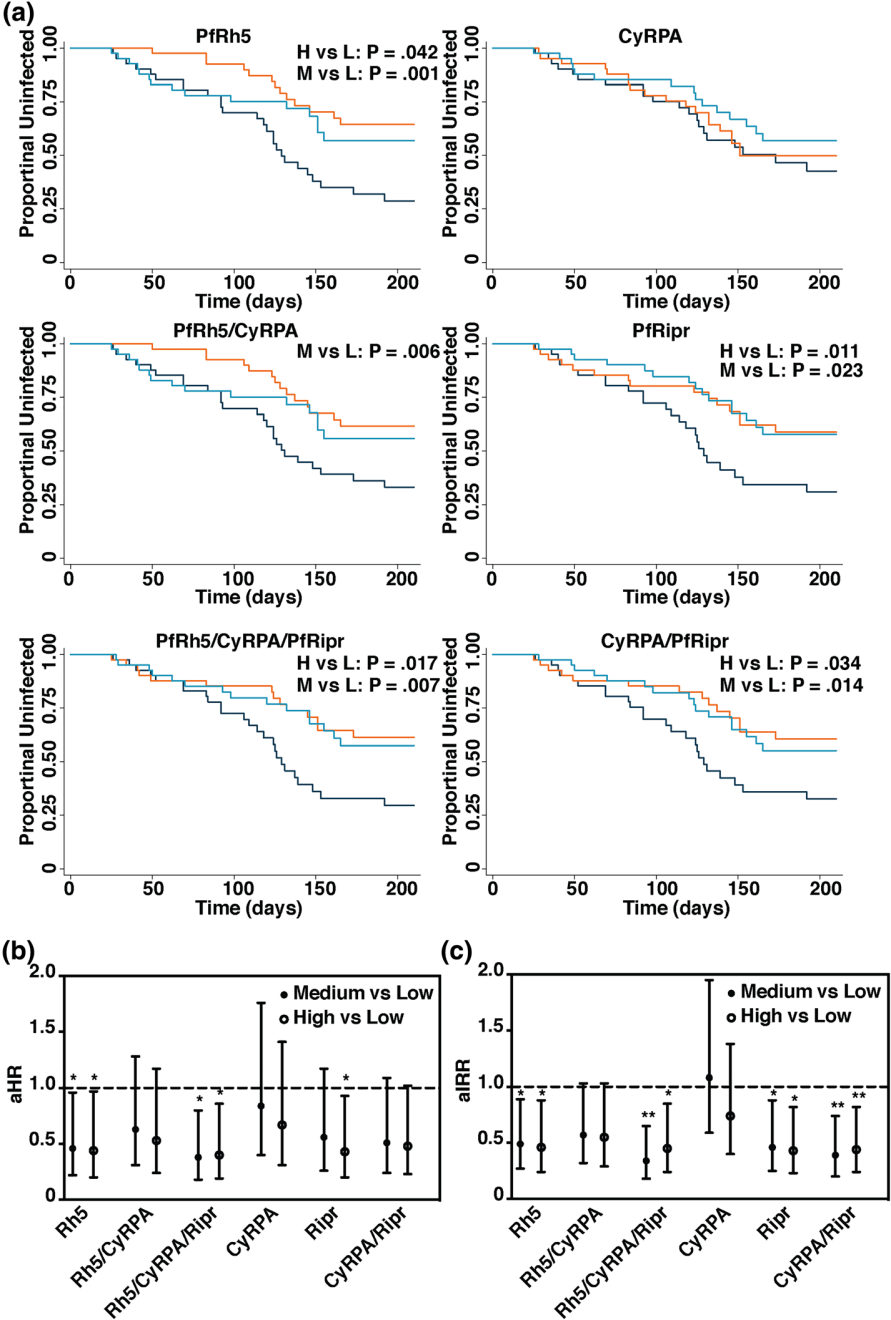
Antibodies to PfRh5 and PfRipr are associated with protection from clinical malaria. (a) Time to first light microscopy positive parasitemia with ≥5,000 parasites/μl in individuals with low (L), medium (M), or high (H) titers of antibody to PfRh5, PfRh5/CyRPA, PfRh5/CyRPA/PfRipr, CyRPA, PfRipr, CyRPA/PfRipr at study enrolment, as determined with Kaplan–Meier survival analysis. (b) Hazard ratios were determined to assess associations of antibody responses with time to first clinical episode and (c) incidence rate ratios were determined to assess associations between antibody responses and incidence of clinical episodes. Age‐adjusted ratios with 95% confidence interval are shown. Clinical episode was defined by the presence of fever plus light microscopy positive parasitemia with ≥5,000 parasites/μl. ^*^ p ≤ .05, ^**^ p ≤ .01

We investigated the association between protection from reinfection with *P. falciparum* malaria and antibody titres in response to PfRh5, CyRPA, and PfRipr, as well as the complexes PfRh5/CyRPA, CyRPA/PfRipr, and PfRh5/CyRPA/PfRipr. We used plasma samples collected from a longitudinal cohort of 206 children living in a malaria‐endemic area of Papua New Guinea (Figure 5). Antibody titres were measured to the recombinant proteins alone or in binary and ternary complexes. Antibody titres were heterogenous and, for analysis, recipients were divided into terciles of low (L)‐, medium (M)‐, and high (H)‐level responders. The time‐to‐reinfection, and first clinical episode and differences between high/medium and low responders, was determined by Kaplan–Meier survival curves with log rank test and Cox proportional hazards models (Figure 5a,b). A Poisson regression model was used to analyse the differences of clinical incidences between groups (Figure 5c). Statistical analysis of the Kaplan–Meier survival curves revealed significant differences between high‐/medium‐level responders and low‐level responders to PfRh5 and PfRipr in time‐to‐reinfection and proportions of individuals remaining malaria‐free, with higher antibody levels associated with less malaria (Figure 5a). High‐ and medium‐level antibody responses to PfRh5 and PfRipr were associated with a significantly lower risk of clinical episode compared with low‐level responses, both in terms of time to first microscopy‐positive blood smears (Figure 5a) and the time‐to‐first clinical episode (Figure 5b) and the incidence of clinical episodes (Figure 5c). Conversely, no significant differences were observed between groups in response to CyRPA; and the CyRPA/PfRipr and PfRh5/CyRPA/PfRipr complexes gave similar responses to those of PfRh5 and PfRipr antigens alone. PfRipr showed stronger associations with protection than Rh5 and adding Rh5 into a multivariate model including both antigens did not significantly improve the associations with protection (data not shown). This finding suggests that higher levels of naturally acquired antibodies to PfRh5 and PfRipr are associated with protection against clinical malaria following natural exposure to *P. falciparum*, whereas even relatively high levels of naturally acquired antibodies against CyRPA are not associated with similar protection.

## 3 DISCUSSION

The PfRh5/CyRPA/PfRipr complex is essential for invasion of merozoites into human erythrocytes (Volz et al., 2016), and consequently these proteins are potential candidates for inclusion in a vaccine against malaria. PfRh5 and CyRPA are under preclinical investigation as vaccine candidates, whereas a role for PfRipr remains unexplored in this respect. The current study was undertaken to examine whether the trimeric protein complex, or PfRipr alone, may be important vaccine candidates that should be further pursued for preclinical development.

A major impediment for developing PfRipr as a potential vaccine component has been its complex, disulphide‐linked domain structure and the inability to express a functional form of the full‐length protein (Chen et al., 2011). Using a Drosophila cell expression system, we have expressed and purified full‐length functional recombinant PfRipr that can form a trimeric complex with PfRh5 and CyRPA. Previously, the same expression system was used to obtain large quantities of functional PfRh5 for vaccine trials (Hjerrild et al., 2016; Jin et al., 2018). Thus, it provides a potential means to produce GMP‐quality PfRipr for testing its potential as a vaccine against malaria in human clinical trials. It could also be a useful tool for further biological studies.

The PfRh5/CyRPA/PfRipr complex as well as PfRh5, PfRipr, and CyRPA alone was used to raise antibodies that were tested for their abilities to inhibit growth of four independent strains of *P. falciparum*. Of the polyclonal antibodies tested here by GIA, those to the C‐terminal portion of PfRipr were the most inhibitory, despite having very similar ELISA reactivity (and thus antibody titre) against Ripr_FL to antibodies raised against this antigen. Unfortunately, this antigen was not suitable for further vaccine development due to its propensity to form highly aggregated multimers (Healer & Cowman, unpublished data). Antibodies raised against full‐length Ripr performed second best in GIA, followed closely by those raised against PfCyRPA. We observed that antibodies to PfRh5 and the PfRh5/CyRPA/PfRipr complex were less inhibitory in these assays. In combinatorial assays using antibodies against Fl‐Ripr, CyRPA, and Rh5, all the more potent combinations included anti‐Fl‐Ripr antibodies. The finding that combinations of mAbs against PfRipr and CyRPA acted synergistically to block *P. falciparum* invasion and growth supports further study to determine whether inclusion of at least two of the antigens present in the invasion complex in an antimalarial vaccine provides better levels of protection than a single antigen.

These results contrasted with the somewhat higher levels of GIA seen in other PfRh5 vaccination studies in rabbits (Bustamante et al., 2013; Douglas et al., 2011). This may arise from the fact that the protein used in this study, despite its ability to bind to basigin (Chen et al., 2014) and form a ternary complex with CyRPA and Ripr, was produced as a truncated protein, lacking the 15 kDa N‐terminal portion. Note that the N‐terminal region of PfRh5 is cleaved from the protein and not required for basigin binding although antibodies against this specific domain were inhibitory in GIA assays (Galaway et al., 2017). Furthermore, it is difficult to directly compare GIA results from different studies using different antibody concentrations and different assay measurements. Note that the N‐terminal region of PfRh5 is cleaved from the protein and not required for basigin binding although antibodies against this specific domain were inhibitory in GIA assays (Galaway et al., 2017). A detailed comparison of the inhibitory effects of each the monoclonal antibodies against Rh5, CyRPA, and Ripr would identify protective epitopes on these proteins and could be used to further inform vaccine design.

A previous study has shown the protein P113 serves as the anchor for this complex on the merozoite surface for binding to basigin by binding to the 15 kDa N‐terminus of PfRh5 (Galaway et al., 2017). Other studies have shown that P113 associates with the PTEX translocon in the parasitophorous vacuole (Elsworth et al., 2016) and its orthologue in *P. berghei* does not function in merozoite invasion (Offeddu, Rauch, Silvie, & Matuschewski, 2014). Additionally, immuno‐precipitation of PfRh5 and the PfRh5/CyRPA/PfRipr complex in *P. falciparum* has not identified any interaction of P113 (Volz et al., 2016). Further data are required to confirm if P113 plays a role in the function of the PfRh5/CyRPA/PfRipr complex in merozoite invasion and the function of the N‐terminal 15 kDa region of PfRh5 and its potential importance in immunity.

Our results show that the PfRh5/CyRPA/PfRipr complex was not a better immunogen in terms of producing more‐potent parasite growth‐inhibitory antibodies compared with PfRh5, PfRipr, or CyRPA alone. The comparative ELISA demonstrated that CyRPA, Rh5, and Ripr were less immunogenic when presented in the 1:1:1 trimeric complex. This difference was most striking for CyRPA, which, after adjusting for relative difference in antigen dose between single and complex immunisation, resulted in approximately 7.5‐fold lower antibody titres when presented in the trimeric complex compared with immunisation as a single immunogen. This finding confirms predictions from the structural model that the inhibitory epitopes on CyRPA are masked by the binding faces of Ripr and Rh5. This was also the case for Rh5 and Ripr but to a lesser degree, perhaps suggesting that there was a level of immune interference between the three antigens when immunised as a complex, although there is a level of inter‐rabbit variation in responsiveness against any antigen so it is difficult to make conclusive statements without further studies to this effect (Elias et al., 2013).

The relatively lower GIA activity seen with the complex IgG also suggests that neoantigens arising from the molecular interactions within the complex are not as important as those accessible to antibody in the individual proteins when it comes to induction of growth‐inhibitory antibodies (in rabbits). Some of the epitopes of neutralising antibodies present in individual antigens are masked or presented differently in the complex.

These results demonstrate that blocking the PfRh5/CyRPA interaction inhibits complex assembly, and this is an important mechanism by which antibodies inhibit merozoite invasion of erythrocytes. In contrast, parasite‐neutralising anti‐Ripr mAbs did not interfere with PfRh5/CyRPA/PfRipr complex formation, suggesting they inhibit merozoite invasion through a different mechanism. The inhibitory epitope of these anti‐PfRipr mAbs is within the EGF 7 domain, suggesting that this region plays an important function other than complex association. Neutralising antibodies to EGF‐7 may act to block insertion of PfRipr into the erythrocyte membrane (Wong et al., 2019). Nevertheless, it is clear that an antibody response to epitopes on the EGF 7 domain of this protein can play an important role in blocking merozoite invasion of *P. falciparum*.

The anti‐CyRPA mAb 8A7 blocks assembly of PfRh5 into the complex whereas 3D1 and 5B12 do not, despite them all binding to common epitopes. Parallel studies of the CyRPA crystal structure determined that monoclonal antibodies 8A7 and C12 in fact bind to proximal but not identical regions of CyRPA, which may explain their differential abilities to block the Rh5‐CyRPA interaction (Chen et al., 2017; Favuzza et al., 2017). Based on cryo‐EM and crosslinking studies (Wong et al., 2019), the C‐terminal tail of Rh5 interacts with blade 1 beta sheet of CyRPA. The CyRPA‐8A7 crystal structure showed that 8A7 binds to blade 1‐2 of CyRPA therefore explaining the ability of 8A7 to inhibit PfRh5‐CyRPA interaction, whereas the CyRPA‐C12 crystal structure showed that C12 binds to blade 2‐3 of CyRPA.

The affinity of binding for the three CyRPA mAbs was very similar, but 8A7 had a much slower dissociation rate compared with 3D1 and 5B12. However, all the three mAbs showed similar levels of inhibition in GIA, suggesting that differences in the kinetic properties of these antibodies were not a factor in binding to and inhibiting parasite invasion. Although 8A7 may interfere with merozoite invasion by blocking of PfRh5 binding to CyRPA, both 3D1 and 5B12 must act via a distinct mechanism such as steric hindrance that interferes with subsequent functions of the PfRh5/CyRPA/PfRipr complex.

Our analysis confirms previous studies showing that naturally acquired antibodies to PfRh5 and PfRipr are significantly associated with protection from malaria in longitudinal cohort studies of children exposed to *P. falciparum* (Chiu et al., 2014; Richards et al., 2013; Tran et al., 2014; Weaver et al., 2016). In contrast, no association with protection was found with antibodies to CyRPA nor to the binary Rh5/CyRPA complex. PfRh5 and PfRipr do not naturally form a complex, except when CyRPA is present (Wong et al., 2019), so this combination was not included in these assays. Antibodies against the CyRPA/Ripr binary complex appeared to show no additional benefit in terms of relative risk of malaria compared with responses against PfRh5 or PfRipr alone.

Antibodies to the ternary complex were also significantly associated with protection. The significant protection that appears to be associated with higher levels of antibodies to the complex may simply reflect the importance of antibodies to Rh5 and Ripr, as it is impossible to determine in these assays what epitopes are relevant to the protective response. The significant association seen with antibodies to the binary CyRPA/Ripr complex suggests this to be the case. However, unequivocal proof that antibodies against Ripr and Rh5 are responsible for clinical protection from malaria would require testing in human clinical trials.

A possible explanation for the apparent discordancy between the observed lack of significant association observed between antibodies to CyRPA acquired by natural exposure to *P. falciparum* and the results presented here with vaccination‐induced antibodies blocking invasion of parasites in vitro is that antibody titres against neutralising epitopes in a natural infection may not reach the level required for a functional protective response. Related to this may be that in *P. falciparum* infections, the antigens are present predominantly in a ternary complex and the key neutralising epitopes on CyRPA are hidden at the interface with Rh5. It is important to note that vaccination with CyRPA alone induced antibodies with growth‐inhibitory activity, and therefore, further studies to examine an immunisation approach that includes uncomplexed CyRPA in combination with other antigens would be of interest.

The lack of significant association between antibodies against CyRPA and protection from *P. falciparum* malaria is consistent with a previous study identifying potential combinatorial antibody associations for *P. falciparum* vaccine development, in that antibodies to CyRPA produced as a full‐length protein in HEK293 cells showed no association with protective immunity in Malian children, whereas IgG raised against the recombinant protein was significantly inhibitory against parasite growth in GIA assays (Bustamante et al., 2017). The authors concluded that PfCyRPA is poorly immunogenic in humans. In contrast to this, antibodies against PvCyRPA were a key component in combinations of antibodies associated with protective immunity against *P. vivax* malaria (Franca et al., 2017). Because these two antigens are closely related, this finding raises interesting questions about their relative immunogenicity in humans with exposure to the different species of *Plasmodium*. Ripr orthologues are found in other *Plasmodium* species including *P. vivax*, so a better understanding of the potential interactions of these proteins could provide deeper insights into their biological role across the genus and their importance as vaccine candidates.

These results support inclusion of PfRipr in future malaria vaccine development programs and suggest that either alone or coimmunized with an effective PfRh5 or CyRPA antigen, Ripr could provide superior protection against blood stage malaria.

## 4 EXPERIMENTAL PROCEDURES

### 4.1 Expression and purification of recombinant proteins

Full‐length PfRipr (amino acids 20–1086) was expressed in *Drosophila* S2 cells (ExpreS^2^ion Biotechnologies) and purified using Strep‐Tactin®XT and size exclusion chromatography (Hjerrild et al., 2016).

A synthetic gene of Ct‐Ripr (encoding amino acids 604–1086; Geneart) was cloned into the insect cell vector pgpHFT (Xu et al., 2010). pgpHFT‐ct‐Ripr was cotransfected with FlashBAC (Oxford Expression Technologies) in Sf21 cells. The seed virus was amplified to obtain high titre virus stocks which were used to induce protein expression in Sf21 cells. The FLAG‐tagged protein was purified by affinity capture on anti‐FLAG M2 agarose resin (Sigma Aldrich). Bound proteins were eluted with FLAG peptide and further purified using size exclusion chromatography.

The EGF1‐2 synthetic gene (encoding amino acids 263–354) was cloned into the pET45b + plasmid with a hexa‐His tag and enterokinase cleavage site at the N‐terminus and transformed into *E. coli* cells for protein expression. The recombinant protein was purified from inclusion bodies and refolded. Further purification steps were performed using a Ni‐nitrilotriacetic acid column (Qiagen) under native conditions, followed by size exclusion chromatography using a Superdex 200 gel filtration column (GE Healthcare Life Sciences).

EGF 6‐8 (encoding 791–900 amino acids) was codon‐optimised for expression in *E. coli* (Genscript) and cloned into the plasmid pET‐45b (+) (Novagen) using *Bam* HI and *Xho* I restriction sites to allow expression of a recombinant protein with an N‐terminal hexa‐His tag. His‐tagged recombinant protein was purified from soluble lysate by affinity purification on Ni‐Sepharose 6 Fast Flow resin (GE Healthcare) and further purified by size exclusion chromatography.

EGF 5‐7 (encoding amino acids 717–860) and EGF 6‐10 (encoding amino acids 816–987) synthetic genes (Geneart) were cloned into the plasmid pPICZαβ (Geneart®) and transformed into *Pichia pastoris*. Proteins were produced in a 10‐L fermentation batch and the culture supernatant diluted with distilled water (fivefold) at pH 4. Cation‐exchange chromatography was carried out on SP‐Sepharose. Size‐exclusion chromatography was utilised as an additional purification step. Where further purification was required, reverse‐phase high‐performance liquid chromatography (RP‐HPLC) was carried out because these disulphide‐rich proteins are resistant to irreversible denaturation under HPLC‐elution conditions.

None of the recombinant protein sequences were modified to replace predicted glycosylation sites.

### 4.2 Immunisations

For generation of polyclonal antisera, rabbits were immunised with 200 μg of antigen in Freund's complete adjuvant and boosted at Days 28 and 56 with the same amount of antigen in Freund's incomplete adjuvant. Serum was prepared from terminal bleeds taken at Day 70 post‐initial immunisation. For polyclonal antisera against the Rh5/CyRPA/Ripr complex, 200 μg of complexed antigens were used as described (Wong et al., 2019). IgG was purified from serum as described previously (Healer et al., 2013). Monoclonal antibodies were generated as previously described (Chen et al., 2017). The animal work was conducted at The Walter and Eliza Hall Institute of Medical Research in accordance with WEHI animal ethics approved protocol AEC 2017.018.

### 4.3 Surface plasmon resonance

Surface plasmon resonance binding assays were performed using a BIAcore 4000 instrument, in SPR buffer as follows: 10mM HEPES, 150mM NaCl, 3.4mM EDTA, 0.005% Tween‐20, pH 7.4. Monoclonal antibodies were immobilised as the ligand on a CM5 sensor chip surface by amine coupling at densities ranging from 1,516 to 5,574 RU. Sensorgrams were double referenced and initially fitted to a Langmuir specific one‐site binding model and then a heterogeneous ligand‐binding model, if appropriate, to derive on and off rates and the dissociation constants (*K*_*D*_). Competitive assays were performed by pre‐incubating the analyte with the varying concentrations (78 to 1250 ng ml^−1^) of the competing antibody with either CyRPA or PfRipr at a constant 8nM prior to flowing over the ligand immobilised surface. Kinetic parameters and competition experiments were derived from three experiments performed on independent days.

### 4.4 Assembly of PfRh5/CyRPA/PfRipr complex and effect of monoclonal antibodies.

The PfRh5/CyRPA/PfRipr complex was assembled by incubating equimolar amounts of proteins in 20mM Tris, pH 8.5, 150mM NaCl for 1 hr at room temperature. To test the effect of monoclonal antibodies on complex assembly, twofold molar excesses of anti‐PfRh5, anti‐CyRPA, or anti‐PfRipr mAbs were incubated with the respective antigens in 20mM Tris, pH 8.5, 150mM NaCl for 1 hr at room temperature. After antigen–antibody complex formation, the remaining components of the PfRh5/CyRPA/PfRipr complex were added to the antigen–antibody complex for 1 hr at room temperature to enable full complex assembly. The mixture was injected into a Superose 6 10/300 size exclusion column in 20mM Tris, pH 8.5, 150mM NaCl. Eluted fractions were separated by nonreducing SDS‐PAGE to determine the effect of monoclonal antibodies on complex assembly.

### 4.5 Growth inhibition assays using purified IgG

Growth inhibition assays were performed as described previously (Healer et al., 2013). Briefly, trophozoite stage parasites at 0.1% parasitemia that were grown in a 50 μl culture at 2% haematocrit in 96 well round bottom microtitre plates (Falcon) with twofold dilutions of each antibody. After incubation for 96 hr, each well was fixed at room temperature for 30 min with 50 μl of 0.25% glutaraldehyde (ProSciTech) diluted in human tonicity PBS. Following centrifugation at 1,200 rpm for 2 min, supernatants were discarded and parasites stained with 50 μl SYBR Green (Invitrogen) diluted in PBS. The parasitemia of each well was determined by counting 50,000 cells by flow cytometry using a Cell Lab Quanta SC–MPL Flow Cytometer (Beckman Coulter). Growth was expressed as a percentage of the parasitemia obtained using a non‐immune IgG control. All samples were tested in triplicate and standard deviation calculated.

GIA with rabbit polyclonal antibodies (results in Figure 4a) were performed at the GIA reference centre, NIAID/NIH, using their protocol (Malkin et al., 2005). In brief, purified IgGs at the concentrations described in the results section were incubated with *P. falciparum*‐infected RBCs for 40 hr at 37°C. The final parasitemia was determined by biochemical measurement of *Pf* lactate dehydrogenase.

### 4.6 Cohort study

Plasma samples were obtained from a treatment to reinfection study of 206 children aged 5–14 performed in Madang of Papua New Guinea (PNG). The details of this cohort have been described previously (Michon et al., 2007). Briefly, participants had oral artesunate treatment at enrolment to clear existing infections. They were actively monitored every 2 weeks for the symptomatic illness and parasitemia during the 6‐month follow‐up period. Reinfection was confirmed by using post‐polymerase chain reaction and ligase detection reaction‐fluorescent microsphere assay and light microscopy with Giemsa‐stained blood smears. This study was approved by the Medical Research Advisory Committee, PNG Ministry of Health, The Walter and Eliza Hall Institute Human Research Ethics Committee. Written consent was obtained from parents or guardians of all participants.

### 4.7 Antigen conjugation and measurement of IgG responses

Conjugation of recombinant proteins to Luminex 5.6 polystyrene microspheres was performed as previously described (Kellar et al., 2001). To ensure the same amount of the combined molecule on the beads for the individual antigens and complex, the corresponding concentrations of each of the six conjugates used to conjugate 2.5 × 10^6^ microspheres were as follows: PfRh5 = 4.8 μg/ml; CyRPA = 3.84 μg/ml; PfRipr = 11.52 μg/ml; PfRh5/CyRPA = 8.64 μg/ml; CyRPA/PfRipr = 15.36 μg/ml; PfRh5/CyRPA/PfRipr = 20.16 μg/ml. Mouse monoclonal antibody only recognising the corresponding PfRh5, CyRPA, and PfRipr were used to confirm the correct conjugation of antigen complex with the beads.

The multiplex antibody detection assay was performed as described elsewhere (Franca et al., 2016; Kellar et al., 2001). Briefly, plasma samples were diluted into 1:100 in phosphate‐buffered saline containing 1% bovine serum albumin and 0.05% Tween (resulting buffer was denoted as PBT). Samples were incubated with the conjugated beads (1:50 ratio) for 30 min under constant agitation. PE‐conjugated donkey anti‐human IgG fragment crystallisable region (Fc) was used as the secondary antibody in a volume of 100 μL/well of 1 μg/mL. Beads were read on a Bio‐Plex200 reader. Results are reported as median fluorescence intensity (MFI). A blank well without plasma was included for determination of the true fluorescence background. As immune responses to pooled serum from immune PNG adults are very strong, in each batch, a twofold serial dilution from 1/50 to 1/25,600 of this pool was included to generate a standard curve to minimise the variation from different plates. The same negative control plasma used in all assays was an anonymous Melbourne donor with no known previous exposure to malaria.

### 4.8 Statistical analysis

Specific standard curves from each Luminex assay plate were used for transformation of MFIs into relative antibody units (expressed as dilution factors that range from 1.95 × 10^−5^ or 1/51,200 to 0.02 or 1/50) using a five parametric logistic regression model as described previously (Franca et al., 2016).

Statistical analyses were performed using STATA version 12 (StataCorp) and R version 3.2.1 (htpp://cran.r‐project.org). The population was categorised into terciles by antibody levels. Kaplan–Meier curve and log‐rank test were utilised to investigate the associations of antibody responses and time to reinfection. Cox proportional hazards models were used to explore associations between antibody responses and time to first clinical episode. Poisson regression was performed to obtain incidence rate ratios (IRRs) for the incidence of clinical episodes throughout the longitudinal cohort. As described elsewhere, age and distance to the sea were adjusted in the above Cox and Poisson model (Chiu et al., 2016).

## CONFLICT OF INTERESTS

The authors declare no competing financial interests.

## AUTHOR CONTRIBUTIONS

JH, WW, and JKT were responsible for Ripr and CyRPA polyclonal and monoclonal antibody development and screening, GIA assays, analysis of complex formation and expression and purification of PfRh5 and recombinant PfRipr proteins and assisted with writing the manuscript. VS, TMMS, TJ, and WAdJ developed the PfRipr expression and purification strategy in Drosophila S2 cells, produced the PfRipr protein and assisted with manuscript preparation. RWB and PEC were responsible for performing plasmon surface resonance experiments, analysis, and interpretation of the data and assisted with the manuscript. KM and CAL performed parasite GIA and analysed the data. CW, ES, and PNB were responsible for the expression and purification of various fragments of PfRipr in *E. coli* and *P. pastoris* and assisted with writing the manuscript. SD supplied the Rh5 mAb. WH, WHT, and IM were responsible for the collection and analysis of the human cohort samples and interpretation of the data and assisted with the manuscript. AFC was responsible for overall project strategy and management, data interpretation, and writing the manuscript.

## Supporting information


**Figure S1.** **SPR sensorgrams showing direct binding of A. Ripr and B. CyRPA to antibodies.** Raw data (coloured lines) and fits (black lines) are shown for experiments using Ripr or CyRPA as the analyte at various concentrations from low to high as indicated by arrows, with the indicated antibodies immobilized on the sensor surface as the ligand. Data are representative of three experiments. C. kinetic parameters for on and off rates (k_a_ and k_d_) and calculated affinity constants (K_D_) with standard deviations from three experiments performed on independent days indicated.
**Figure S2. Ripr Antibody SPR competition assays.** SPR sensor surfaces were immobilized with A. 1G12 or 5G6, B. 4A8 or 3C3 using a fixed concentration of Ripr as the analyte, preincubated with either 0, 78, 156, 313, 625 or 1250 ng/mL of the competing antibody as the analyte. Curves show a reduction in response upon increasing antibody concentration indicating competing epitopes.
**Figure. S3. Control PfRipr antibody SPR competition assays.** SPR sensor surfaces were immobilized with either 4A8, 3C3 competing with 1G12, or 1G12, 5G6 competing with 3C3. Experiments were performed using a fixed concentration of PfRipr as the analyte, preincubated with either 0, 78, 156, 313, 625 or 1250 ng/mL of the competing antibody as the analyte. Curves show a consistent response even at high concentrations of competing antibody indicating non‐competitive epitopes.
**Figure S4. CyRPA Antibody SPR competition assays.** SPR sensor surfaces were immobilized with 8A7, 5B12 or 3D1 antibodies using a fixed concentration of CyRPA as the analyte, preincubated with either 0, 78, 156, 313, 625 or 1250 ng/mL of the competing antibody as the analyte. Curves either show A. a reduction in response upon increasing antibody concentration indicating competing epitopes or B. no reduction in response indicating non‐completive epitopes.
**Figure S5. Monoclonal antibodies against PfRipr and CyRPA have no effect on PfRh5/CyRPA/PfRipr complex formation.** A. SEC analysis of non‐inhibitory mAb 1C4, which binds to PfRipr, showing it does not block PfRh5/CyRPA/PfRipr complex formation. B. SEC analysis of inhibitory mAb 1G12, which binds to PfRipr but does not block PfRh5/CyRPA/PfRipr complex formation. C. SEC analysis of inhibitory monoclonal antibody 5B12 which binds to CyRPA but does not block PfRh5/CyRPA/PfRipr complex formation. Proteins in all SDS‐PAGE gels are stained with Coomassie blue. Traces shown below SEC gel profiles in all panels give elution volumes of molecular weight standards in kDa.
**Figure S6 Immunisation with individual antigens results in higher titres than immunisation with the trimeric complex.** Comparative ELISA showing the differential responsiveness to the individual antigens when immunised as single immunogens at 200 Δg per dose or as part of a trimeric complex at 200 Δg per dose. Horizontal line designates the serum titres at 1 ELISA Unit (EU) and the perpendicular lines descending from this line show the serum dilution resulting in a value of 1EU for the respective antigens. Specific antigens detected were Ripr_FL (A), CyRPA (B) and Rh5 (C). Titres of antigen‐specific antibody were lower in the complex sera than for individual immunogens. Titres against Ripr antigens Ct and FL were the same (D).
**Table S1.** Summary of SPR data for monoclonal antibody binding to PfRipr and CyRPA.
Note: yellow refers to the monoclonal pairs that show competitive inhibition. This shows: 1G12 and 5G8 have overlapping epitopes for Ripr, 4A8 and 3C3 have overlapping epitopes for Ripr, 8A7, 5B12 and 3D1 have overlapping epitopes for CyRPA.

## References

[cmi13030-bib-0001] Baum, J. , Chen, L. , Healer, J. , Lopaticki, S. , Boyle, M. , Triglia, T. , … Cowman, A. F. (2009). Reticulocyte‐binding protein homologue 5—An essential adhesin involved in invasion of human erythrocytes by *Plasmodium falciparum* . Int. J. Parasitol., 39, 371–380. 10.1016/j.ijpara.2008.10.006 19000690

[cmi13030-bib-0002] Bustamante, L. Y. , Bartholdson, S. J. , Crosnier, C. , Campos, M. G. , Wanaguru, M. , Nguon, C. , … Rayner, J. C. (2013). A full‐length recombinant *Plasmodium falciparum* PfRH5 protein induces inhibitory antibodies that are effective across common PfRH5 genetic variants. Vaccine, 31, 373–379. 10.1016/j.vaccine.2012.10.106 23146673 PMC3538003

[cmi13030-bib-0003] Bustamante, L. Y. , Powell, G. T. , Lin, Y. C. , Macklin, M. D. , Cross, N. , Kemp, A. , … Rayner, J. C. (2017). Synergistic malaria vaccine combinations identified by systematic antigen screening. Proc. Natl. Acad. Sci. U S A, 114, 12045–12050. 10.1073/pnas.1702944114 29078270 PMC5692528

[cmi13030-bib-0004] Chen, L. , Lopaticki, S. , Riglar, D. T. , Dekiwadia, C. , Uboldi, A. D. , Tham, W. H. , … Cowman, A. F. (2011). An EGF‐like protein forms a complex with PfRh5 and is required for invasion of human erythrocytes by *Plasmodium falciparum* . PLoS Pathog, 7, e1002199 10.1371/journal.ppat.1002199 21909261 PMC3164636

[cmi13030-bib-0005] Chen, L. , Xu, Y. , Healer, J. , Thompson, J. K. , Smith, B. J. , Lawrence, M. C. , & Cowman, A. F. (2014). Crystal structure of PfRh5, an essential *P. falciparum* ligand for invasion of human erythrocytes. Elife, 3 10.7554/eLife.04187 PMC4356141 25296023

[cmi13030-bib-0006] Chen, L. , Xu, Y. , Wong, W. , Thompson, J. K. , Healer, J. , Goddard‐Borger, E. , et al. (2017). Structural basis for inhibition of erythrocyte invasion by antibodies to *Plasmodium falciparum* protein CyRPA. Elife, 6 10.7554/eLife.21347 PMC5349848 28195530

[cmi13030-bib-0007] Chiu, C. Y. , Healer, J. , Thompson, J. K. , Chen, L. , Kaul, A. , Savergave, L. , … Mueller, I. (2014). Association of antibodies to *Plasmodium falciparum* reticulocyte binding protein homolog 5 with protection from clinical malaria. Front Microbiol, 5, 314. 25071730 10.3389/fmicb.2014.00314 PMC4074990

[cmi13030-bib-0008] Chiu, C. Y. , White, M. T. , Healer, J. , Thompson, J. K. , Siba, P. M. , Mueller, I. , … Hansen, D. S. (2016). Different regions of *Plasmodium falciparum* Erythrocyte binding antigen‐175 induce antibody responses to infection of varied efficacy. J. Infect. Dis, 214, 96–104. 10.1093/infdis/jiw119 27020092

[cmi13030-bib-0009] Conway, D. J. (2015). Paths to a malaria vaccine illuminated by parasite genomics. Trends Genet, 31, 97–107. 25620796 10.1016/j.tig.2014.12.005 PMC4359294

[cmi13030-bib-0010] Crosnier, C. , Bustamante, L. Y. , Bartholdson, S. J. , Bei, A. K. , Theron, M. , Uchikawa, M. , … Wright, G. J. (2011). Basigin is a receptor essential for erythrocyte invasion by *Plasmodium falciparum* . Nature, 480, 534–537. 10.1038/nature10606 22080952 PMC3245779

[cmi13030-bib-0011] Douglas, A. D. , Baldeviano, G. C. , Lucas, C. M. , Lugo‐Roman, L. A. , Crosnier, C. , Bartholdson, S. J. , … Draper, S. J. (2015). A PfRH5‐based vaccine is efficacious against heterologous strain blood‐stage *Plasmodium falciparum* infection in aotus monkeys. Cell Host Microbe, 17, 130–139. 10.1016/j.chom.2014.11.017 25590760 PMC4297294

[cmi13030-bib-0012] Douglas, A. D. , Williams, A. R. , Illingworth, J. J. , Kamuyu, G. , Biswas, S. , Goodman, A. L. , … Draper, S. J. (2011). The blood‐stage malaria antigen PfRH5 is susceptible to vaccine‐inducible cross‐strain neutralizing antibody. Nat. Commun., 2, 601 10.1038/ncomms1615 22186897 PMC3504505

[cmi13030-bib-0013] Douglas, A. D. , Williams, A. R. , Knuepfer, E. , Illingworth, J. J. , Furze, J. M. , Crosnier, C. , … Draper, S. J. (2014). Neutralization of *Plasmodium falciparum* merozoites by antibodies against PfRH5. J. Immunol., 192, 245–258. 10.4049/jimmunol.1302045 24293631 PMC3872115

[cmi13030-bib-0014] Dreyer, A. M. , Matile, H. , Papastogiannidis, P. , Kamber, J. , Favuzza, P. , Voss, T. S. , … Pluschke, G. (2012). Passive immunoprotection of *Plasmodium falciparum*‐infected mice designates the CyRPA as candidate malaria vaccine antigen. J. Immunol., 188, 6225–6237. 10.4049/jimmunol.1103177 22593616

[cmi13030-bib-0015] Elias, S. C. , Collins, K. A. , Halstead, F. D. , Choudhary, P. , Bliss, C. M. , Ewer, K. J. , … Draper, S. J. (2013). Assessment of immune interference, antagonism, and diversion following human immunization with biallelic blood‐stage malaria viral‐vectored vaccines and controlled malaria infection. J. Immunol., 190, 1135–1147. 10.4049/jimmunol.1201455 23293353 PMC3672846

[cmi13030-bib-0016] Elsworth, B. , Sanders, P. R. , Nebl, T. , Batinovic, S. , Kalanon, M. , Nie, C. Q. , … Gilson, P. R. (2016). Proteomic analysis reveals novel proteins associated with the *Plasmodium* protein exporter PTEX and a loss of complex stability upon truncation of the core PTEX component, PTEX150. Cell Microbiol, 18, 1551–1569. 10.1111/cmi.12596 27019089

[cmi13030-bib-0017] Favuzza, P. , Guffart, E. , Tamborrini, M. , Scherer, B. , Dreyer, A. M. , Rufer, A. C. , … Rudolph, M. G. (2017). Structure of the malaria vaccine candidate antigen CyRPA and its complex with a parasite invasion inhibitory antibody. Elife, 6 10.7554/eLife.20383 PMC5349852 28195038

[cmi13030-bib-0018] Franca, C. T. , Hostetler, J. B. , Sharma, S. , White, M. T. , Lin, E. , Kiniboro, B. , … King, C. L. (2016). An antibody screen of a *Plasmodium vivax* antigen library identifies novel merozoite proteins associated with clinical protection. PLoS Negl. Trop. Dis., 10, e0004639 10.1371/journal.pntd.0004639 27182597 PMC4868274

[cmi13030-bib-0019] Franca, C. T. , White, M. T. , He, W. Q. , Hostetler, J. B. , Brewster, J. , Frato, G. , … Kiniboro, B. (2017). Identification of highly‐protective combinations of *Plasmodium vivax* recombinant proteins for vaccine development. Elife, 6 10.7554/eLife.28673 PMC5655538 28949293

[cmi13030-bib-0020] Galaway, F. , Drought, L. G. , Fala, M. , Cross, N. , Kemp, A. C. , Rayner, J. C. , & Wright, G. J. (2017). P113 is a merozoite surface protein that binds the N terminus of *Plasmodium falciparum* RH5. Nat. Commun., 8, 14333. 28186186 10.1038/ncomms14333 PMC5309799

[cmi13030-bib-0021] Greenwood, B. , & Doumbo, O. K. (2016). Implementation of the malaria candidate vaccine RTS,S/AS01. Lancet, 387, 318–319. 26549465 10.1016/S0140-6736(15)00807-7

[cmi13030-bib-0022] Healer, J. , Thompson, J. K. , Riglar, D. T. , Wilson, D. W. , Chiu, Y. H. , Miura, K. , … Baum, J. (2013). Vaccination with conserved regions of erythrocyte‐binding antigens induces neutralizing antibodies against multiple strains of *Plasmodium falciparum* . PLoS One, 8, e72504 10.1371/journal.pone.0072504 24039774 PMC3769340

[cmi13030-bib-0023] Hjerrild, K. A. , Jin, J. , Wright, K. E. , Brown, R. E. , Marshall, J. M. , Labbe, G. M. , … Illingworth, J. J. (2016). Production of full‐length soluble *Plasmodium falciparum* RH5 protein vaccine using a *Drosophila melanogaster* Schneider 2 stable cell line system. Sci. Rep., 6, 30357 10.1038/srep30357 27457156 PMC4960544

[cmi13030-bib-0024] Jin, J. , Tarrant, R. D. , Bolam, E. J. , Angell‐Manning, P. , Soegaard, M. , Pattinson, D. J. , … Draper, S. J. (2018). Production, quality control, stability, and potency of cGMP‐produced *Plasmodium falciparum* RH5.1 protein vaccine expressed in Drosophila S2 cells. NPJ Vaccines, 3, 32 10.1038/s41541-018-0071-7 30131879 PMC6098134

[cmi13030-bib-0025] Kellar, K. L. , Kalwar, R. R. , Dubois, K. A. , Crouse, D. , Chafin, W. D. , & Kane, B. E. (2001). Multiplexed fluorescent bead‐based immunoassays for quantitation of human cytokines in serum and culture supernatants. Cytometry, 45, 27–36. 10.1002/1097-0320(20010901)45:1<27::AID-CYTO1141>3.0.CO;2-I 11598944

[cmi13030-bib-0026] Malkin, E. M. , Diemert, D. J. , McArthur, J. H. , Perreault, J. R. , Miles, A. P. , Giersing, B. K. , … Durbin, A. P. (2005). Phase 1 clinical trial of apical membrane antigen 1: An asexual blood‐stage vaccine for *Plasmodium falciparum* malaria. Infect. Immun., 73, 3677–3685. 10.1128/IAI.73.6.3677-3685.2005 15908397 PMC1111886

[cmi13030-bib-0027] Michon, P. , Cole‐Tobian, J. L. , Dabod, E. , Schoepflin, S. , Igu, J. , Susapu, M. , … Schofield, L. (2007). The risk of malarial infections and disease in Papua New Guinean children. Am. J. Trop. Med., 76, 997–1008. 10.4269/ajtmh.2007.76.997 PMC3740942 17556601

[cmi13030-bib-0028] Moorthy, V. S. , Newman, R. D. , & Okwo‐Bele, J. M. (2013). Malaria vaccine technology roadmap. Lancet, 382, 1700–1701. 10.1016/S0140-6736(13)62238-2 24239252

[cmi13030-bib-0029] Offeddu, V. , Rauch, M. , Silvie, O. , & Matuschewski, K. (2014). The *Plasmodium* protein P113 supports efficient sporozoite to liver stage conversion in vivo. Mol. Biochem. Parasitol., 193, 101–109. 24657782 10.1016/j.molbiopara.2014.03.002

[cmi13030-bib-0030] Patel, S. D. , Ahouidi, A. D. , Bei, A. K. , Dieye, T. N. , Mboup, S. , Harrison, S. C. , & Duraisingh, M. T. (2013). *Plasmodium falciparum* merozoite surface antigen, PfRH5, elicits detectable levels of invasion‐inhibiting antibodies in humans. J. Infect. Dis., 208, 1679–1687. 23904294 10.1093/infdis/jit385 PMC3805239

[cmi13030-bib-0031] Payne, R. O. , Silk, S. E. , Elias, S. C. , Miura, K. , Diouf, A. , Galaway, F. , … Draper, S. J. (2017). Human vaccination against RH5 induces neutralizing antimalarial antibodies that inhibit RH5 invasion complex interactions. JCI Insight, 2 10.1172/jci.insight.96381 PMC5752323 29093263

[cmi13030-bib-0032] Reddy, K. S. , Amlabu, E. , Pandey, A. K. , Mitra, P. , Chauhan, V. S. , & Gaur, D. (2015). Multiprotein complex between the GPI‐anchored CyRPA with PfRH5 and PfRipr is crucial for *Plasmodium falciparum* erythrocyte invasion. Proc Natl Acad Sci U S A, 112, 1179–1184. 25583518 10.1073/pnas.1415466112 PMC4313826

[cmi13030-bib-0033] Reddy, K. S. , Pandey, A. K. , Singh, H. , Sahar, T. , Emmanuel, A. , Chitnis, C. E. , … Gaur, D. (2014). Bacterially expressed full‐length recombinant *Plasmodium falciparum* RH5 protein binds erythrocytes and elicits potent strain‐transcending parasite‐neutralizing antibodies. Infect. Immun., 82, 152–164. 10.1128/IAI.00970-13 24126527 PMC3911863

[cmi13030-bib-0034] Richards, J. S. , Arumugam, T. U. , Reiling, L. , Healer, J. , Hodder, A. N. , Fowkes, F. J. , … Thompson, J. K. (2013). Identification and prioritization of merozoite antigens as targets of protective human immunity to *Plasmodium falciparum* malaria for vaccine and biomarker development. J. Immunol., 191, 795–809. 10.4049/jimmunol.1300778 23776179 PMC3702023

[cmi13030-bib-0035] RTS,S Clinical Trials Partnership1 , Agnandji, S. T. , Lell, B. , Fernandes, J. F. , Abossolo, B. P. , Methogo, B. G. , … Vansadia, P. (2012). A phase 3 trial of RTS,S/AS01 malaria vaccine in African infants. N. Engl. J. Med., 367, 2284–2295. 23136909 10.1056/NEJMoa1208394 PMC10915853

[cmi13030-bib-0036] Tran, T. M. , Ongoiba, A. , Coursen, J. , Crosnier, C. , Diouf, A. , Huang, C. Y. , … Crompton, P. D. (2014). Naturally acquired antibodies specific for *Plasmodium falciparum* reticulocyte‐binding protein homologue 5 inhibit parasite growth and predict protection from malaria. J. Infect. Dis., 209, 789–798. 10.1093/infdis/jit553 24133188 PMC3923542

[cmi13030-bib-0037] Volz, J. C. , Yap, A. , Sisquella, X. , Thompson, J. K. , Lim, N. T. , Whitehead, L. W. , … Nebl, T. (2016). Essential role of the PfRh5/PfRipr/CyRPA complex during *Plasmodium falciparum* invasion of erythrocytes. Cell Host Microbe, 20, 60–71. 10.1016/j.chom.2016.06.004 27374406

[cmi13030-bib-0038] Weaver, R. , Reiling, L. , Feng, G. , Drew, D. R. , Mueller, I. , Siba, P. M. , … Beeson, J. G. (2016). The association between naturally acquired IgG subclass specific antibodies to the PfRH5 invasion complex and protection from *Plasmodium falciparum* malaria. Sci Rep, 6, 33094 10.1038/srep33094 27604417 PMC5015043

[cmi13030-bib-0039] Weiss, G. E. , Gilson, P. R. , Taechalertpaisarn, T. , Tham, W. H. , de Jong, N. W. , Harvey, K. L. , … Cowman, A. F. (2015). Revealing the sequence and resulting cellular morphology of receptor‐ligand interactions during *Plasmodium falciparum* invasion of erythrocytes. PLoS Pathog, 11, e1004670 10.1371/journal.ppat.1004670 25723550 PMC4344246

[cmi13030-bib-0040] WHO (2016). World Malaria Report. http://www.who.int/malaria/publications/world‐malaria‐report‐2016/en/.

[cmi13030-bib-0041] Williams, A. R. , Douglas, A. D. , Miura, K. , Illingworth, J. J. , Choudhary, P. , Murungi, L. M. , … Draper, S. J. (2012). Enhancing blockade of *Plasmodium falciparum* erythrocyte invasion: Assessing combinations of antibodies against PfRH5 and other merozoite antigens. PLoS Pathog, 8, e1002991 10.1371/journal.ppat.1002991 23144611 PMC3493472

[cmi13030-bib-0042] Wong, W. , Huang, R. , Menant, S. , Hong, C. , Sandow, J. J. , Birkinshaw, R. W. , … Cowman, A. F. (2019). Structure of *Plasmodium falciparum* Rh5‐CyRPA‐Ripr invasion complex. Nature, 565, 118–121. 10.1038/s41586-018-0779-6 30542156

[cmi13030-bib-0043] Wright, K. E. , Hjerrild, K. A. , Bartlett, J. , Douglas, A. D. , Jin, J. , Brown, R. E. , … Higgins, M. K. (2014). Structure of malaria invasion protein RH5 with erythrocyte basigin and blocking antibodies. Nature, 515, 427–430. 10.1038/nature13715 25132548 PMC4240730

[cmi13030-bib-0044] Xu, Y. , Kershaw, N. J. , Luo, C. S. , Soo, P. , Pocock, M. J. , Czabotar, P. E. , … Zhang, J. G. (2010). Crystal structure of the entire ectodomain of gp130: Insights into the molecular assembly of the tall cytokine receptor complexes. J. Biol. Chem., 285, 21214–21218. 10.1074/jbc.C110.129502 20489211 PMC2898449

