# Supplementary material for: Neutralising antibodies block the function of Rh5/Ripr/CyRPA complex during invasion of Plasmodium falciparum into human erythrocytes
Source: Cell Microbiol. 2019 Apr 24;21(7):e13030. doi: 10.1111/cmi.13030 (PMC6594224; doi:10.1111/cmi.13030)
Supplement: Supplementary file 1 — Figure S1. SPR sensorgrams showing direct binding of A. Ripr and B. CyRPA to antibodies. Raw data (coloured lines) and fits (black lines) are shown for experiments using Ripr or CyRPA as the analyte at various concentrations from low to high as indicated by arrows, with the indicated antibodies immobilized on the sensor surface as the ligand. Data are representative of three experiments. C. kinetic parameters for on and off rates (k a and k d) and calculated affinity constants (K D) with standard deviations from three experiments performed on independent days indicated. Figure S2. Ripr Antibody SPR competition assays. SPR sensor surfaces were immobilized with A. 1G12 or 5G6, B. 4A8 or 3C3 using a fixed concentration of Ripr as the analyte, preincubated with either 0, 78, 156, 313, 625 or 1250 ng/mL of the competing antibody as the analyte. Curves show a reduction in response upon increasing antibody concentration indicating competing epitopes. Figure. S3. Control PfRipr antibody SPR competition assays. SPR sensor surfaces were immobilized with either 4A8, 3C3 competing with 1G12, or 1G12, 5G6 competing with 3C3. Experiments were performed using a fixed concentration of PfRipr as the analyte, preincubated with either 0, 78, 156, 313, 625 or 1250 ng/mL of the competing antibody as the analyte. Curves show a consistent response even at high concentrations of competing antibody indicating non‐competitive epitopes. Figure S4. CyRPA Antibody SPR competition assays. SPR sensor surfaces were immobilized with 8A7, 5B12 or 3D1 antibodies using a fixed concentration of CyRPA as the analyte, preincubated with either 0, 78, 156, 313, 625 or 1250 ng/mL of the competing antibody as the analyte. Curves either show A. a reduction in response upon increasing antibody concentration indicating competing epitopes or B. no reduction in response indicating non‐completive epitopes. Figure S5. Monoclonal antibodies against PfRipr and CyRPA have no effect on PfRh5/CyRPA/PfRipr complex format [file CMI-21-na-s001.pdf]

A

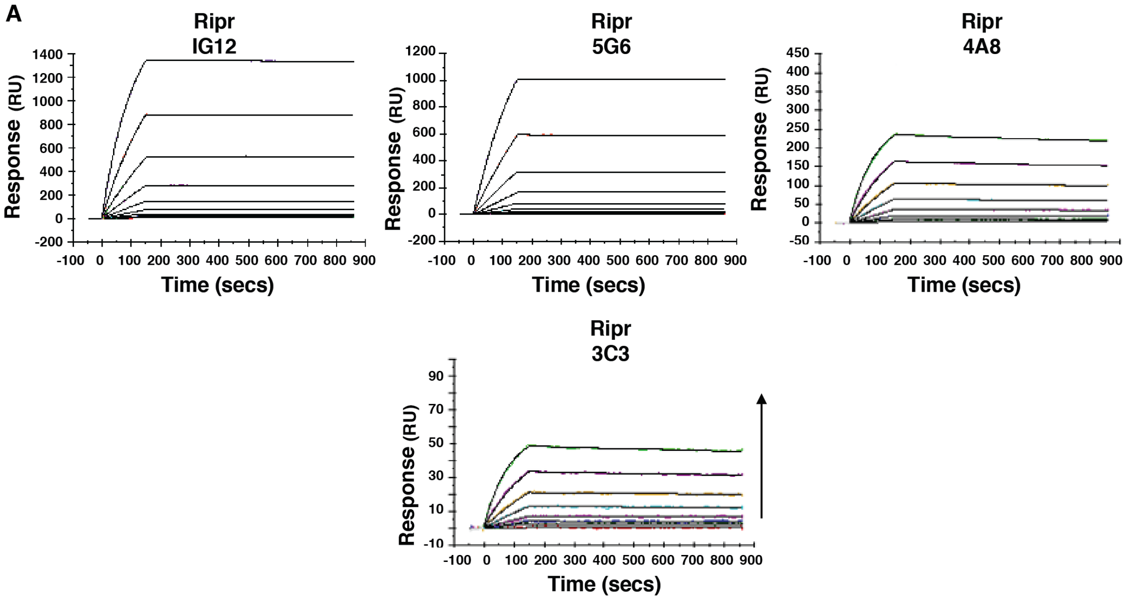

B

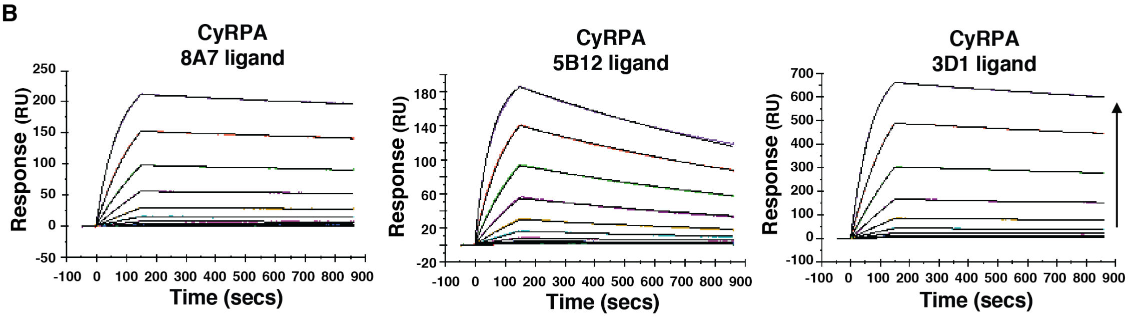

C

		k_a (M^{-1} s^{-1})	k_d (s^{-1})	K_D (M)
anti-Ripr mAb	1G12	1.18 (+/- 0.01) x 10^5	4.7 (+/-1.9) x 10^{-6}	4.0 (+/-1.6) x 10^{-11}
	5G6	1.08 (+/- 0.41) x 10^5	6.7 (+/-2.4) x 10^{-6}	6.4 (+/-0.9) x 10^{-11}
	4A8	4.6 (+/- 1.3) x 10^5	1.06 (+/-0.13) x 10^{-4}	2.4 (+/-0.6) x 10^{-10}
	3C3	4.6 (+/- 1.3) x 10^5	9.2 (+/-1.3) x 10^{-5}	2.1 (+/-0.7) x 10^{-10}
anti-CyRPA mAb	8A7	1.85 (+/- 0.12) x 10^5	9.1 (+/- 3.7) x 10^{-5}	5.7 (+/- 2.0) x 10^{-10}
	5B12	2.16 (+/- 0.04) x 10^5	5.9 (+/- 1.6) x 10^{-4}	2.7 (+/- 0.3) x 10^{-9}
	3D1	1.96 (+/- 0.4) x 10^5	1.28 (+/- 0.37) x 10^{-4}	6.5 (+/- 0.6) x 10^{-10}

Fig. S1. SPR sensorgrams showing direct binding of A. Ripr and B. CyRPA to antibodies. Raw data (coloured lines) and fits (black lines) are shown for experiments using Ripr or CyRPA as the analyte at various concentrations from low to high as indicated by arrows, with the indicated antibodies immobilized on the sensor surface as the ligand. Data are representative of three experiments. C. kinetic parameters for on and off rates (k_a and k_d) and calculated

affinity constants (K_D) with standard deviations from three experiments performed on independent days indicated.

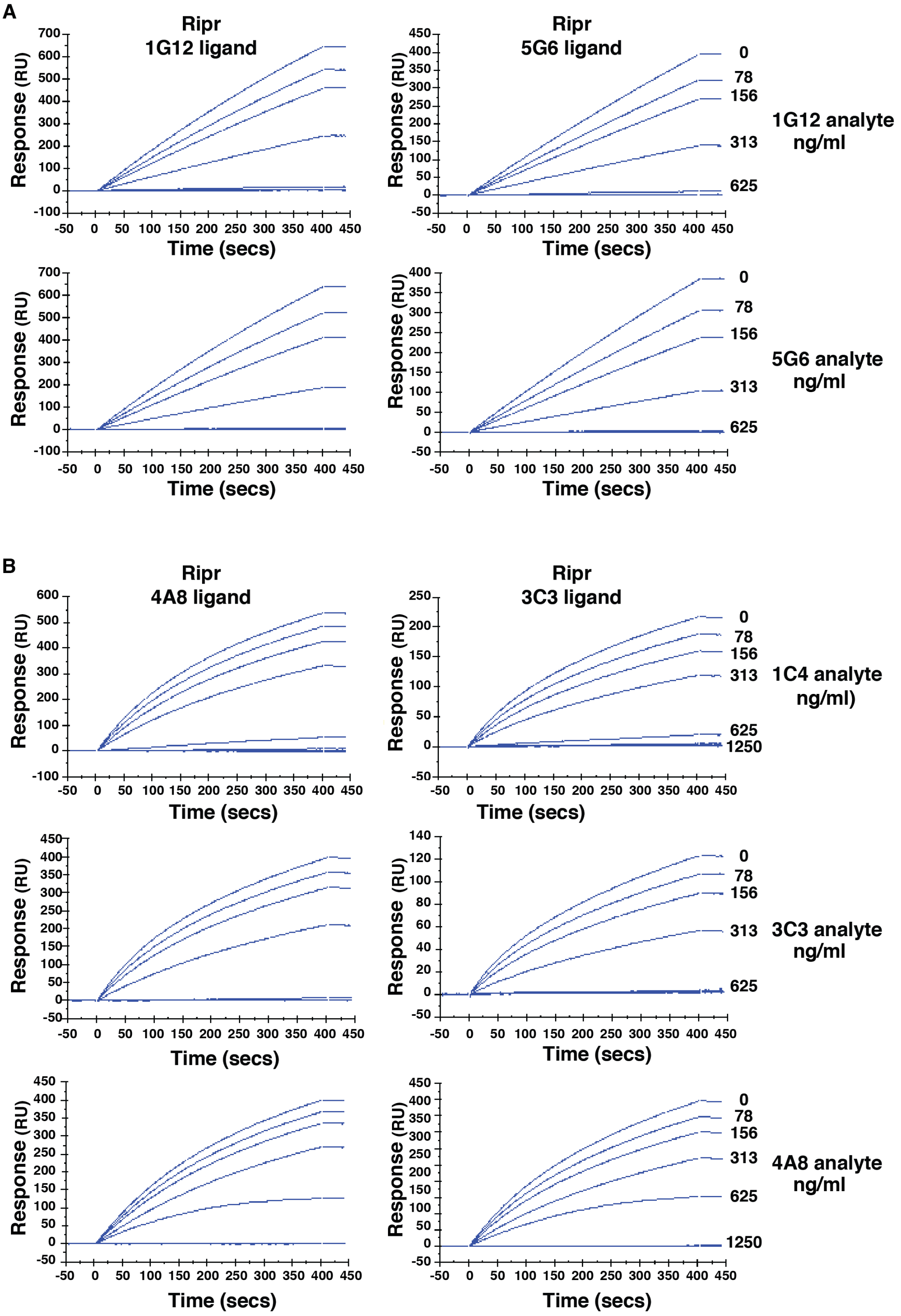

Fig. S2. Ripr Antibody SPR competition assays. SPR sensor surfaces were immobilized with A. 1G12 or 5G6, B. 4A8 or 3C3 using a fixed concentration of Ripr as the analyte, preincubated with either 0, 78, 156, 313, 625 or 1250 ng/mL of the competing antibody as the analyte. Curves

show a reduction in response upon increasing antibody concentration indicating competing epitopes.

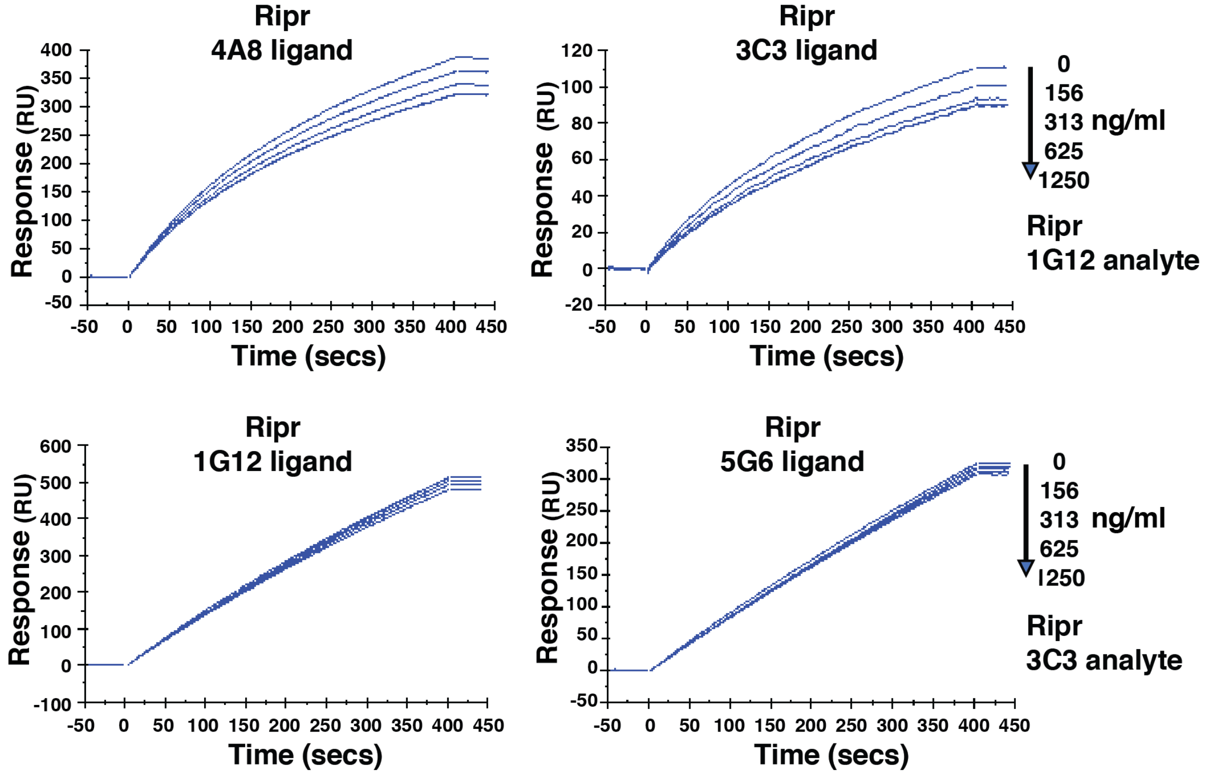

Fig. S3. Control PfRipr antibody SPR competition assays. SPR sensor surfaces were immobilized with either 4A8, 3C3 competing with 1G12, or 1G12, 5G6 competing with 3C3. Experiments were performed using a fixed concentration of PfRipr as the analyte, preincubated with either 0, 78, 156, 313, 625 or 1250 ng/mL of the competing antibody as the analyte. Curves show a consistent response even at high concentrations of competing antibody indicating non-competitive epitopes.

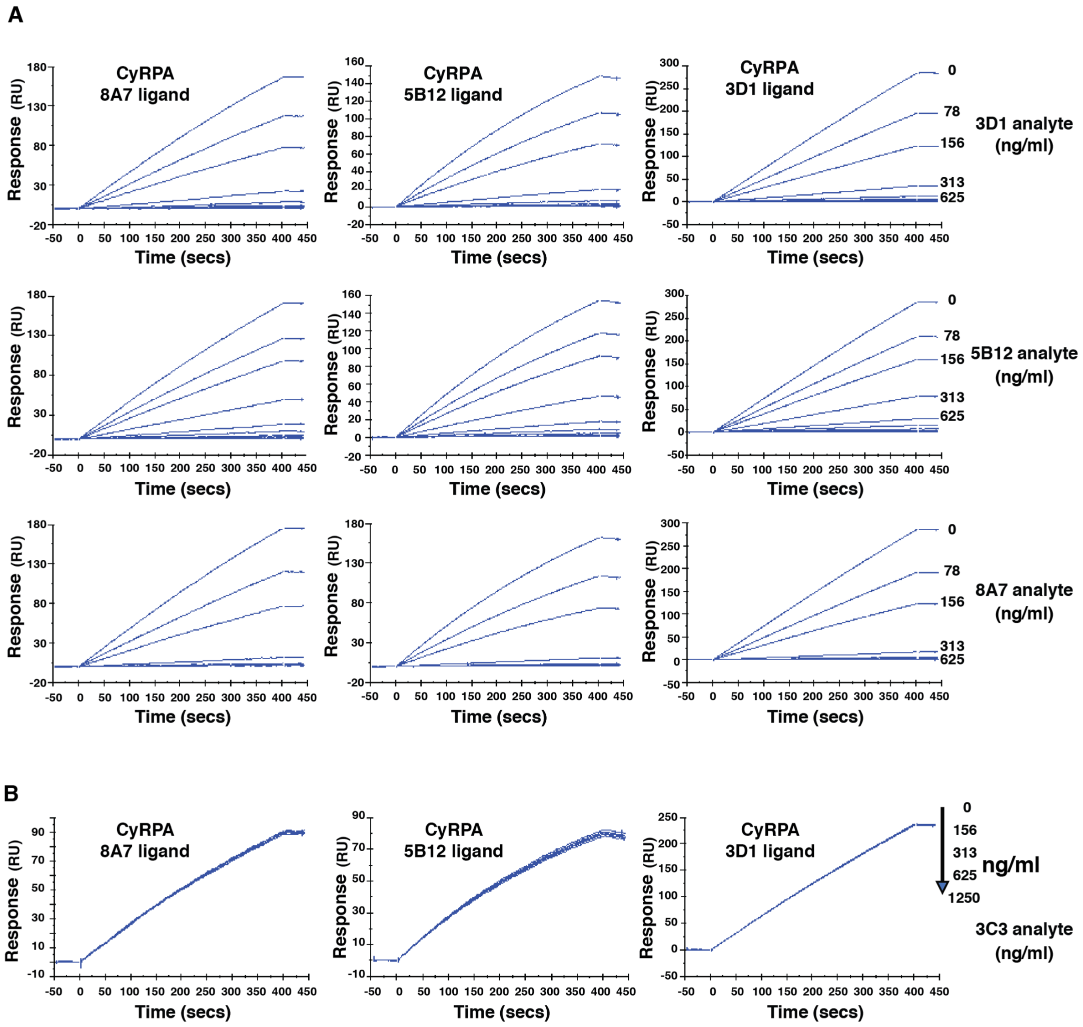

Fig. S4. CyRPA Antibody SPR competition assays. SPR sensor surfaces were immobilized with 8A7, 5B12 or 3D1 antibodies using a fixed concentration of CyRPA as the analyte, preincubated with either 0, 78, 156, 313, 625 or 1250 ng/mL of the competing antibody as the analyte. Curves either show A. a reduction in response upon increasing antibody concentration indicating competing epitopes or B. no reduction in response indicating non-completive epitopes.

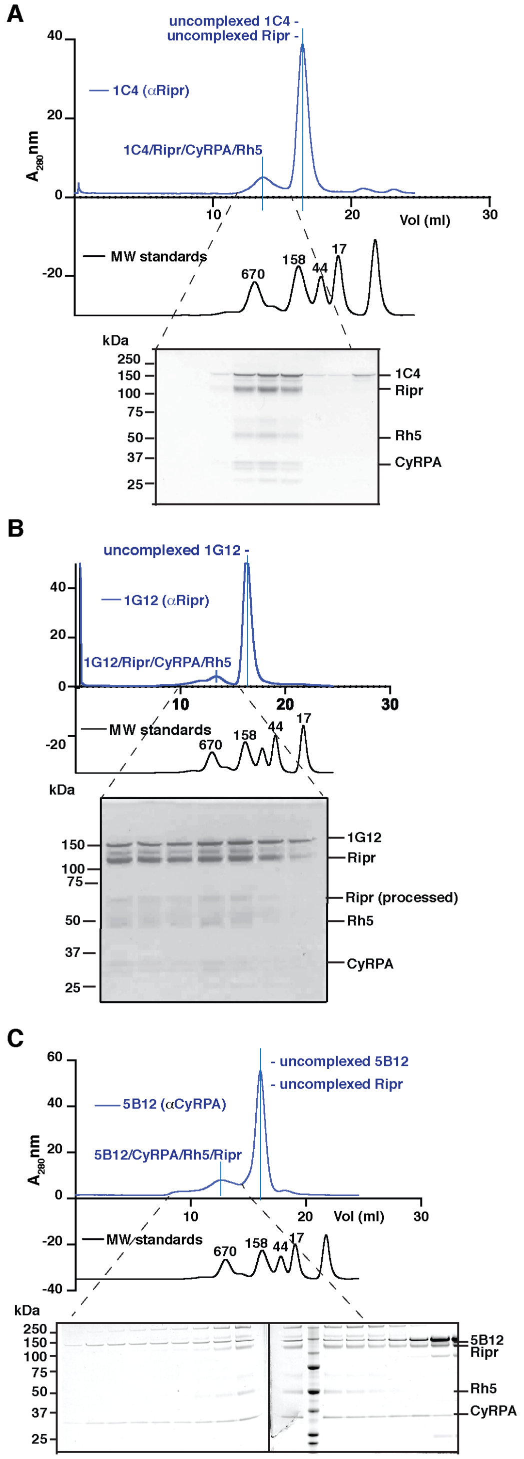

Fig. S5. Monoclonal antibodies against PfRipr and CyRPA have no effect on PfRh5/CyRPA/PfRipr complex formation. A. SEC analysis of non-inhibitory mAb 1C4, which binds to PfRipr, showing it does not block PfRh5/CyRPA/PfRipr complex formation.

B. SEC analysis of inhibitory mAb 1G12, which binds to PfRipr but does not block PfRh5/CyRPA/PfRipr complex formation. C. SEC analysis of inhibitory monoclonal antibody 5B12 which binds to CyRPA but does not block PfRh5/CyRPA/PfRipr complex formation. Proteins in all SDS-PAGE gels are stained with Coomassie blue. Traces shown below SEC gel profiles in all panels give elution volumes of molecular weight standards in kDa.

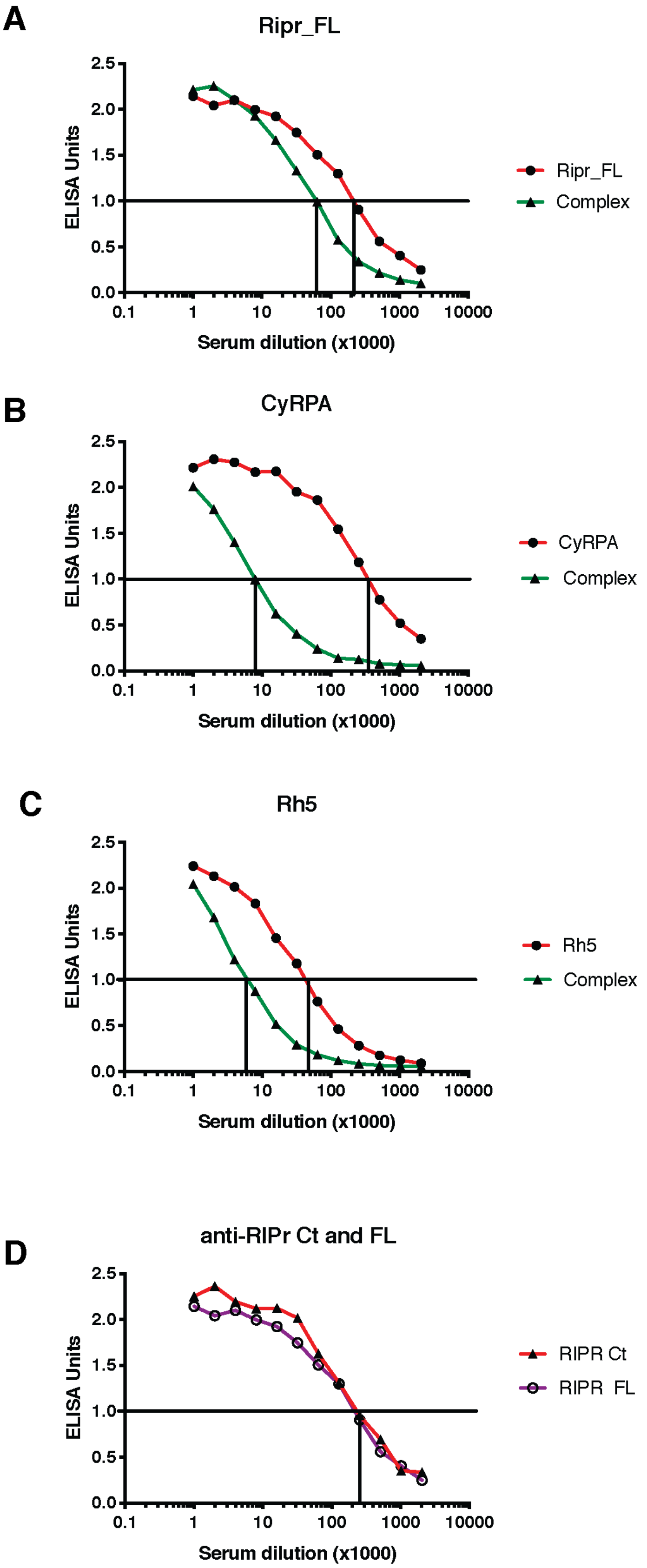

Fig. S6 Immunisation with individual antigens results in higher titres than immunisation with the trimeric complex. Comparative ELISA showing the differential responsiveness to the individual antigens when immunised as single immunogens at 200 µg per dose or as part

of a trimeric complex at 200 µg per dose. Horizontal line designates the serum titres at 1 ELISA Unit (EU) and the perpendicular lines descending from this line show the serum dilution resulting in a value of 1EU for the respective antigens. Specific antigens detected were Ripr_FL (A), CyRPA (B) and Rh5 (C). Titres of antigen-specific antibody were lower in the complex sera than for individual immunogens. Titres against Ripr antigens Ct and FL were the same (D).

Supplementary Table 1. Summary of SPR data for monoclonal antibody binding to PfRipr and CyRPA.

			Analyte						
			anti-Ripr mAb				anti-CyRPA mAb		
			1G12	5G6	4A8	3C3	8A7	5B12	3D1
Ligand	anti-Ripr mAb	1G12	Yes	Yes	ND	No	No	ND	ND
		5G6	Yes	Yes	ND	No	No	ND	ND
		4A8	No	ND	Yes	Yes	No	ND	ND
		3C3	No	ND	Yes	Yes	No	ND	ND
	anti-CyRPA mAb	8A7	No	ND	ND	No	Yes	Yes	Yes
		5B12	No	ND	ND	No	Yes	Yes	Yes
		3D1	No	ND	ND	No	Yes	Yes	Yes

Note: yellow refers to the monoclonal pairs that show competitive inhibition. This shows: 1G12 and 5G8 have overlapping epitopes for Ripr, 4A8 and 3C3 have overlapping epitopes for Ripr, 8A7, 5B12 and 3D1 have overlapping epitopes for CyRPA.
